# Structural and DNA end resection study of the bacterial NurA-HerA complex

**DOI:** 10.1186/s12915-023-01542-0

**Published:** 2023-02-24

**Authors:** Jieyu Yang, Yiyang Sun, Ying Wang, Wanshan Hao, Kaiying Cheng

**Affiliations:** 1 Key Laboratory of Aging and Cancer Biology of Zhejiang Province, Department of Immunology and Pathogen Biology, School of Basic Medical Sciences, Hangzhou Normal University, Hangzhou, 311121 China; 2 State Key Laboratory for Diagnosis and Treatment of Infectious Diseases, The First Affiliated Hospital, College of Medicine, Zhejiang University, Hangzhou, 310003 China

**Keywords:** *Deinococcus*, DNA end resection, Nuclease, NurA-HerA, Translocase

## Abstract

**Background:**

The nuclease NurA and the ATPase/translocase HerA play a vital role in repair of double-strand breaks (DSB) during the homologous recombination in archaea. A NurA-HerA complex is known to mediate DSB DNA end resection, leading to formation of a free 3′ end used to search for the homologous sequence. Despite the structures of individual archaeal types of NurA and HerA having been reported, there is limited information regarding the molecular mechanisms underlying this process. Some bacteria also possess homologs of NurA and HerA; however, the bacterial type of this complex, as well as the detailed mechanisms underlying the joining of NurA-HerA in DSB DNA end resection, remains unclear.

**Results:**

We report for the first time the crystal structures of *Deinococcus radiodurans* HerA (drHerA) in the nucleotide-free and ADP-binding modes. A *D. radiodurans* NurA-HerA complex structure was constructed according to a low-resolution cryo-electron microscopy map. We performed site-directed mutagenesis to map the drNurA-HerA interaction sites, suggesting that their interaction is mainly mediated by ionic links, in contrast to previously characterized archaeal NurA-HerA interactions. The key residues responsible for the DNA translocation activity, DNA unwinding activity, and catalytic activities of the drNurA-HerA complex were identified. A HerA/FtsK-specific translocation-related motif (TR motif) that guarantees the processivity of double-stranded DNA (dsDNA) translocation was identified. Moreover, a mechanism for the translocation-regulated resection of the 5′ tail of broken dsDNA and the corresponding generation of a recombinogenic 3′ single-stranded DNA tail by the drNurA-HerA complex was elucidated.

**Conclusions:**

Our work provides new insights into the mechanism underlying bacterial NurA-HerA-mediated DSB DNA end resection, and the way this complex digests the 5′ tail of a DNA duplex and provides long 3′ free end for strand invasion in the bacterial homologous recombination process.

**Supplementary Information:**

The online version contains supplementary material available at 10.1186/s12915-023-01542-0.

## Background

Homologous recombination (HR) plays an important role in double-stranded break (DSB) repair in an error-free manner, with a widely conserved mechanism in all domains of life [1, 2]. The first event required for the repair of DSB via HR is the broken DNA end resection, which is the resection of the broken DNA end by a coupled nuclease-helicase [1]. The resultant long 3′ free end is used to search the homologous sequence by recombinase [1].

Archaea utilizes NurA and HerA, which functionally cooperate with Mre11 and Rad50, to promote broken DNA end resection [3–6]. HerA is a circular ATP-dependent bipolar translocase and helicase [3, 7, 8]; it belongs to the HerA/FtsK bacterial translocases family, an offshoot of the RecA family [9]. Structural study of *Sulfolobus solfataricus* HerA (ssoHerA, PDB ID: 4D2I) revealed a hexameric ring structure with a central pore well-suited to accommodate the double-stranded DNA (dsDNA) [7]. However, an abundance of the heptameric form of ssoHerA was also detected in the solution, especially in the absence of nucleotides, which provides a more accessible channel to dsDNA and acts as a DNA loading intermediate [10]. NurA was reported to display Mn^2+^-dependent 5′-3′ exonuclease/endonuclease activities on both single-stranded DNA (ssDNA) and dsDNA [3, 11, 12]. Crystallography studies of *S. solfataricus* NurA (ssoNurA, PDB ID: 2YGK) and *Pyrococcus furiousus* NurA (pfNurA, PDB ID: 3TAI) identified a toroidal dimer structure with two RNaseH-like catalytic cores [11, 13]. The dAMP-Mn^2+^-bound pfNurA structure (PDB ID: 3TAZ) provided a possible interpretation for the 5′–3′ directional nuclease activity of NurA [11]. Both structural and biochemical data indicated that archaeal HerA and NurA physically interact at a 6:2 molar ratio, which is mainly mediated by hydrophobic interactions [7, 14]. Low-resolution cryo-electron microscopy (cryo-EM) data of the ssoNurA-HerA complex suggested that HerA is a dsDNA translocase that feeds DNA into the NurA nuclease sites [14]. The narrow channels formed by the NurA dimer were supposed to accommodate the melted DNA duplex [11, 13]. However, the structural features for melting the DNA duplex have not been identified on the archaeal NurA-HerA complex. The dsDNA unwinding and translocation mechanisms are not well-characterized. The catalytic activities of NurA and HerA are mutually interdependent, in a manner not fully understood. Furthermore, the regulation mechanisms for this complex preferably digesting the 5′ tail of a DNA duplex and providing a recombinogenic 3′ ssDNA tail for strand invasion in a cooperative manner are still unclear.

The broken DNA end resection process in bacteria is mediated by the RecBCD/AddAB or the RecJ-RecQ nuclease-helicase complex [15–17]. Bacteria from *Deinococcus-Thermus* phylum which lack the RecBCD/AddAB system, mainly employ RecJ, a 5′ end ssDNA exonuclease, together with a certain helicase, such as RecQ, for efficient broken DNA end processing [16, 18]. The robust DNA damage response and repair ability of *Deinococcus radiodurans* are mainly due to its efficient RecFOR homologous recombination repair system and an extended synthesis-dependent strand annealing (ESDSA) process prior to DNA recombination [19–21]. It was reported that the RecJ-mediated broken DNA end processing is important in both RecFOR and ESDSA pathways [19–21]. The genes encoding NurA and HerA proteins were also identified in bacteria, such as those from the *Deinococcus-Thermus* phylum [22]. Protein sequence identities between archaeal NurA-HerA and bacterial NurA-HerA were less than 20% [22]. The biological functions of bacterial NurA-HerA remain controversial, considering that the deletion of *nurA* or *herA* gene exhibit modest or improved resistance to gamma irradiation, ultraviolet (UV) radiation, and mitomycin C (MMC) treatment in *D. radiodurans* or *Thermus thermophiles* [22–24]. The structural and biochemical characterizations of bacterial NurA-HerA are not yet fully studied. The first bacterial NurA structure from *Thermotoga maritima* (tmNurA, PDB ID: 1ZUP) has been deposited in the protein structure database but with little characterization. The negatively stained assembly of *T. thermophilus* HerA (ttHerA) was performed by electron microscopy (EM), and ttHerA was observed as a hexameric ring-like structure [24]. However, detailed structural and biochemical information of bacterial NurA-HerA to interpret the mechanisms of its DNA end resection process is not yet available.

Herein, we characterized, for the first time, the crystal structures of *D. radiodurans* HerA (drHerA), with and without ADP binding, and a low-resolution cryo-EM structure of the drNurA-HerA complex. The structure features between bacterial NurA-HerA and archaeal NurA-HerA were compared and discussed. We performed site-directed mutagenesis to map the drNurA-HerA interaction sites and to identify the key residues on drNurA-HerA that were involved in DNA translocation, DNA unwinding, and catalysis. We observed that the DNA end resection is mediated by drNurA-HerA in a translocation-regulated mechanism and that the 5′ tail of the dsDNA can be digested during translocation, while the 3′ tail of the dsDNA can only be digested when translocation just initiated or paused. We also found that drNurA-HerA harbors an ATP-dependent endonuclease activity on the 5′ tail of a dsDNA in a distance-specific cutting pattern. This is probably due to the regular conformational changes of the NurA catalytic site, which is influenced by the translocation cycle of drHerA.

## Results

### The structures of drHerA in nucleotide-free and ADP-binding modes

Full-length drHerA without any ligand was crystallized in space group P3_1_21 and the apo structure was solved at 3.0 Å resolution by single-wavelength anomalous diffraction (SAD) phasing using selenomethionine-substituted protein crystals and then refined with the native data. The refined drHerA apo structure contains one hexamer (six protomers) in the asymmetric unit. The drHerA-ADP complex was crystallized in space group P2_1_2_1_2_1_, and the structure was solved at 3.4 Å resolution by the molecular replacement method using drHerA apo structure as the search model. The refined drHerA-ADP complex model contains two hexamers (twelve protomers) in the asymmetric unit; each protomer is bound to one ADP molecule. The crystallographic statistics are given in Table 1.

**Table 1:** Statistics from crystallographic analysis

Complex	Se-HerA	HerA	HerA-ADP
PDB ID	-	7WRW	7WRX
**Data collection**			
Source	SSRF-BL10U2	SSRF-BL02U1	SSRF-BL02U1
Wavelength (Å)	0.9762 (peak)	1	0.9795
Resolution (Å)	49.51-3.1 (3.15-3.1)	49.41-3.0 (3.05-3.0)	102.96-3.4 (3.46-3.4)
Space group	P3_1_21	P3_1_21	P2_1_2_1_2_1_
Cell dimensions: a, b, c	279.84, 279.84, 105.87	279.03, 279.03, 105.68	106.01, 202.87, 411.85
Observations	872275 (40532)	581681 (28756)	644347 (30229)
Unique reflections	86205 (4280)	94205 (4621)	120020 (5712)
R_merge_ (%)	9.6 (86.9)	8.3 (83.5)	15.7 (61.8)
I/σI	19.1 (3.2	16.4 (2.6)	7.8 (2.8)
Completeness (%)	100 (99.8)	99.8 (99.1)	97.5 (94.1)
Redundancy/Anom Redundancy	10.1/5.2	6.2/-	5.4/-
**Refinement statistics**			
Resolution (Å)	-	10.0-3.0 (3.05-3.0)	10.0-3.4 (3.46-3.4)
R_factor_ (%)/R_free_ (%)	-	22.5/24.6	27.6/29.1
rmsd bonds (Å)/angles (°)	-	0.010/1.279	0.008/1.24
Ramachandran plot: Outliers (%)/Favored (%)	-	0/97.2	0/96.4

The values in parentheses refer to the last shell

R_factor_ = Σ||F(obs)- F(calc)||/Σ|F(obs)|

R_free_ = R factor calculated using 5.0% of the reflection data randomly chosen and omitted from the start of refinement

**Fig. 1.**
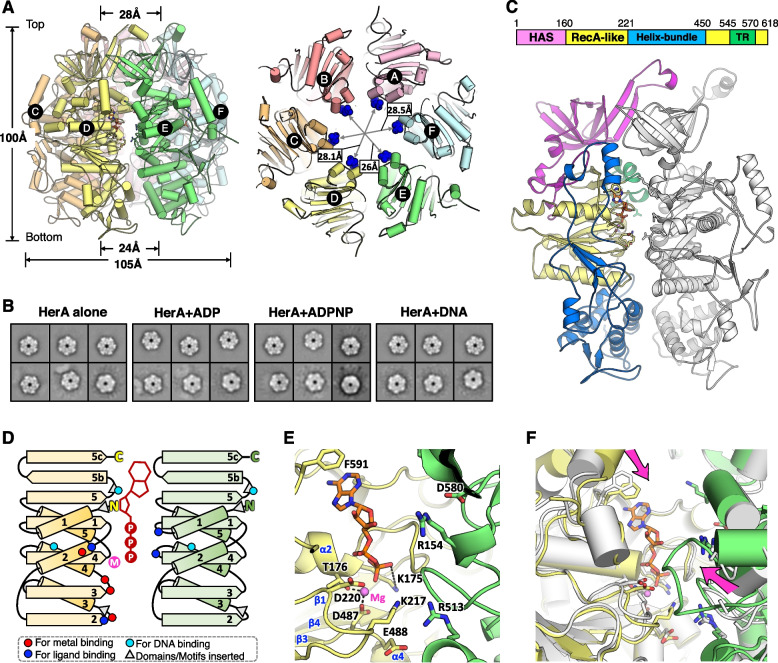
Analysis of the folding, hexamerization, and ligand binding of drHerA. **A** The side and top view of the drHerA-ADP hexamer. Each subunit is labeled and highlighted in distinct colors. In the top view of drHerA, the HAS domains were hidden in order to show the DNA-binding residues (six Arg495 are shown as the surface with blue color) clearly. The size of the drHerA-ADP complex and the diameter of the ring were measured in PyMOL and are labeled. **B** 2D class averages from cryo-EM showed that the drHerA exists as a hexamer, with or without the addition of ligand/dsDNA. **C** Details of the drHerA protomer. Upper, the domain arrangement of drHerA. The HAS, RecA-like, and helix-bundle domains are colored magenta, yellow, and marine, respectively. The motifs important for DNA translocation are colored lime green. Lower, a cartoon view of the drHerA protomer. Each domain is colored the same as the domain arrangement. The neighboring protomer is colored white. **D** The topology diagram of drHerA RecA-like domain. Residues for metal binding, ATP binding, and DNA binding are shown as red, blue, and cyan dots, respectively. Inserted domains/motifs are shown as grey triangles. **E** The zoom-in view of the ATP catalytic sites. The residues important for ligand and metal binding are shown as sticks and labeled. **F** Overview of the conformational changes of the ATP catalytic sites between the apo drHerA structure and the drHerA-ADP complex structure. The two structures were superimposed on the RecA-like domain, Chain D. The HerA apo structure is colored white. Arrows indicate directions of the movement of structural elements

Not as apo ssoHerA that predominantly exists as a heptamer in solution [10], drHerA in both nucleotide-free and ADP-binding modes have been crystallized as sixfold symmetrical rings (Fig. 1A; Additional file 1: Fig. S1). The 2D classification results from low-resolution cryo-EM data also indicated that drHerA forms stable hexamers, regardless of the presence or absence of a nucleotide (ADP or ADPNP) or dsDNA substrate (46bp, without ligand) (Fig. 1B). The overall structure of drHerA-ADP has a height of 100 Å and its outside diameter is approximately 105 Å. Interestingly, the hexamer displays a certain asymmetry, with ~26–28.5 Å inside diameter of the putative DNA translocation motifs, ~23–25.5 Å inside diameter of the putative DNA entrance, and ~27.5–29 Å inside diameter of the putative DNA exit. Such asymmetry is probably due to the differences of ADP-binding states or simply because of crystal packing. The shape of the nucleotide-free drHerA hexamer is quite similar to the drHerA-ADP complex, except that the central pore formed by the six subunits is highly symmetric, with a ~28.2 Å inside diameter of the putative DNA translocation motifs, ~24.5 Å inside diameter of the putative DNA entrance, and ~28.5 Å inside diameter of the putative DNA exit (Additional file 1: Fig. S1). Overall, the shape of drHerA is slightly bigger than that of the ssoHerA hexamer, including the outside and inside diameters, and therefore can provide a more accessible channel to dsDNA than that of the ssoHerA hexamer.

Common to all additional strand catalytic “E” family (ASCE) P-loop ATPases, the ATP catalytic site is formed at the junction between two drHerA protomers. Each drHerA protomer comprises three domains: the N-terminal HerA-ATP synthase barrel domain (HAS-barrel domain) that forms the putative exit for the substrate, the RecA-like ATPase domain, and the helix-bundle domain which is inserted into the RecA-like ATPase domain and forms the putative entrance for the substrate (Fig. 1C). There is also a HerA/FtsK-specific translocation-related motif (TR motif) inserted into the RecA-like ATPase domain, which consists of short helices that are linked by several loops (Fig. 1C; Additional file 2: Fig. S2).

As a canonical RecA-like subgroup member from the ASCE family, the RecA-like ATPase domain of drHerA consists of a conserved ASCE core, in which five stranded β sheets (51432, ↑↑↑↑↑) are sandwiched between a series of α-helices (α1-α5) [9] (Fig. 1C, D). Two extra β sheets (β5b and β5c) fused with the C-terminus of β5 were also observed, which is a unique structural feature of the RecA-like subgroup members (Fig. 1C, D).

Although ADP•AlF_3_ was used as an ATP analog for crystallization, the density of aluminum fluoride, which represents the γ-phosphate position in the transition state, is missing in the density map. The residue Lys217 and the trans-acting residue Arg513, as well as Arg154 in the neighboring subunit, which is supposed to bind the γ-phosphate group of ATP molecule, are located quite far away from the ligand in this structure (Fig. 1E). Therefore, this ligand-binding structure was considered as a drHerA-ADP complex. The binding of ADP resulted in each RecA-like domain to move closer to the neighboring RecA-like domain (Fig. 1F), generating a more tightened hexamer but still a more-or-less flat ring. We believe that the binding of ATP analog (ADP•AlF_3_) will cause different conformational changes and make the drHerA-ADP•AlF_3_ complex fail to be crystalized in this condition. The Walker-A motif is located between strands β1 and α1 (Fig. 1D). In this motif, the conserved lysine (K175) forms ion pairs with the β-phosphate and the threonine residue (T176) coordinates a Mg^2+^ ion (Fig. 1E). The Walker-B motif is located between strands β3 and α4 (Fig. 1D), where the conserved acidic residues (D487 and E488) further coordinate the Mg^2+^ ion (Fig. 1E). The catalytic glutamate (D220) that gives its name to the ASCE superfamily is located at the C-terminus of β2 (Fig. 1D). Previous work showed that mutations of K175 and D487/E488 on drHerA impaired both the ATPase activity of drHerA and the DNA resection by the drNurA-HerA complex [22].

The six N-terminal HAS-barrel domains from the hexamer formed a pore with an approximately 28.5 Å diameter that is large enough for B-form dsDNA threading (Fig. 1C). Similar to ssoHerA, the HAS-barrel domain of drHerA mediates the interaction with drNurA, which will be discussed below.

The inserted motifs/domains between β2 and α3 of the ASCE core formed the helix-bundle domain, which protrudes from the RecA-like ATPase domain (Fig. 1C, D). A pore with an approximately 24.5 Å diameter was formed by the helix-bundle domains, which is enough for the entry of the B-form dsDNA. Unlike ssoHerA, the helix-bundle domain of drHerA is not only formed by helices but also a pair of β sheets with uncharacterized functions. Furthermore, the residues 220–260 on the drHerA helix-bundle protrude into the HAS-barrel domain and partially block the ATP binding site. In contrast, the ATP binding sites in ssoHerA are highly solvent-accessible (Additional file 2: Fig. S2A) [22]. Whether such blocks on drHerA will impair the ATP binding or ADP releasing remains unknown (Fig. 1C). The C-terminal protrusion of ssoHerA was reported to embrace the adjacent protomer and stabilize the hexamer [7]; however, this may not be the case for drHerA. Although the last fourteen residues of the C-terminal protrusion are disordered in our drHerA structures, we believe they are too short to stretch into the neighboring protomer. In addition, the longer α2 on the drHerA RecA-like domain might provide an extra steric hindrance for the binding of the adjacent C-terminal protrusion (Fig. 1C).

### Analysis of drNurA dimerization

**Fig. 2.**
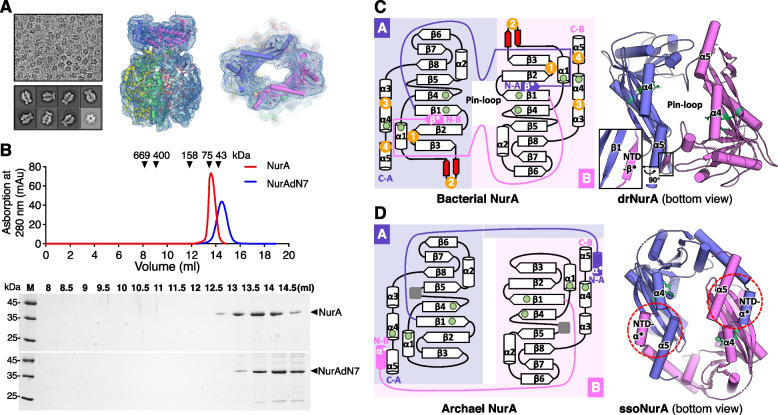
Analysis of the folding and dimerization of drNurA. **A** The overview of the cryo-EM analysis of drNurA-HerA. Upper left, an example of a typical micrograph showing particle distribution of the drHerA-NurA complex. Lower left, a selection of 2D class averages representing different particle views. Middle, the side view of the model of the drNurA-HerA complex fitted into the cryo-EM map. Right, the top view of the model of the drNurA-HerA complex fitted into the cryo-EM map. **B** Gel filtration analysis of the dimerization of drNurA. Wild-type drNurA and drNurA with β* (the N-terminal seven residues) deleted mutant (drNurAdN7) were analyzed with Superdex S200 HR 10/300 column. Peaks of each injection were reconstructed using GraphPad Prism software. Fractions eluted were resolved by 12% SDS-PAGE. **C** Left, the topology diagram of bacterial NurA. The catalytic sites are shown as green dots. The potential drNurA-HerA interaction motifs are numbered and colored orange. The inserted motif (a β-turn) in bacterial NurA is colored red. Right, the bottom view of drNurA dimer. The catalytic sites are labeled as sticks and colored green. The dimerization area on drNurA is squared and the zoom-in view from another orientation is shown by the side. **D** Left, the topology diagram of archaeal NurA. The inserted motifs are shown as grey squares. Right, the bottom view of the ssoNurA (PDB ID: 2YGK) dimer

To study the drNurA-HerA complex, cryo-EM analysis was performed (Fig. 2A). A low-resolution (~9 Å) electron density map was obtained according to image stacks collected from a 200-kV cryo-transmission electron microscope. Because we failed to crystalize drNurA, the initial drNurA model was predicted by the AlphaFold2 online protein structure prediction tool [25]. The drHerA apo structure and the predicted drNurA structure were further organized and fitted into the low-resolution cryo-EM map of the drHerA-drNurA complex by Chimera [26] (Fig. 2A). Little changes on HerA Cα backbone were made after refinement. The Cα backbone of drNurA was further manually refined in COOT according to the real density of the EM map (Fig. 2A). During the revision of this manuscript, another group published a higher resolution (~3.9 Å) cryo-EM structure of the apo drHerA-NurA complex (PDB ID: 7F6D), using a Titan Krios electron microscope (Thermo Fisher) operating at 300 kV with a Gatan K2 Summit detector [27]. Considering good fitness was observed when comparing these two cryo-EM structures (the root-mean-square deviation value for 3160 Cα atoms is 1.355 Å; Additional file 3: Fig. S3), we believe that the Cα backbone of drNurA we built and the overall structure of drNurA-HerA we provided for analysis below are reliable.

Both the bacterial-type NurA and the archaeal-type NurA share conserved RNaseH-like catalytic domains, which are dominated by eight β-strands (67854123, ↓↑↓↑↑↑↓↑), with one α-helix (α1) inserted between β3 and β4, one α-helix (α2) inserted between β6 and β5, and three α-helices (α3, α4, and α5) located at the C-terminus (Fig. 2C, D). However, there are distinct features between them. Firstly, the bacterial-type NurA shows a different dimerization form compared with the archaeal-type NurA. For the archaeal-type NurA, the region before β1 folds into one α-helix (α*) and protrudes into the hydrophobic pocket formed by β2, β3, α4, and α5 of the second NurA molecule (Fig. 2D). For the bacterial-type NurA, the region before β1 folds into one β-strand (β*), which is anti-parallelly located with β1 of the second NurA protomer (Fig. 2C). Deletion of the first seven residues of drNurA (drNurAdN7) disrupted its dimerization (Fig. 2B). Secondly, the bacterial-type NurA has an extra insertion motif between β3 and α1, which tends to form a β-turn and may assist in drHerA binding (that will be discussed below) (Fig. 2C). Finally, the bacterial-type NurA lacks the extra motif between β4 and β5, which comprises a mixture of α-helices and β-strands and contributes to the dimerization of pfNurA [11].

### Mapping of the drNurA-HerA interaction sites

**Fig. 3.**
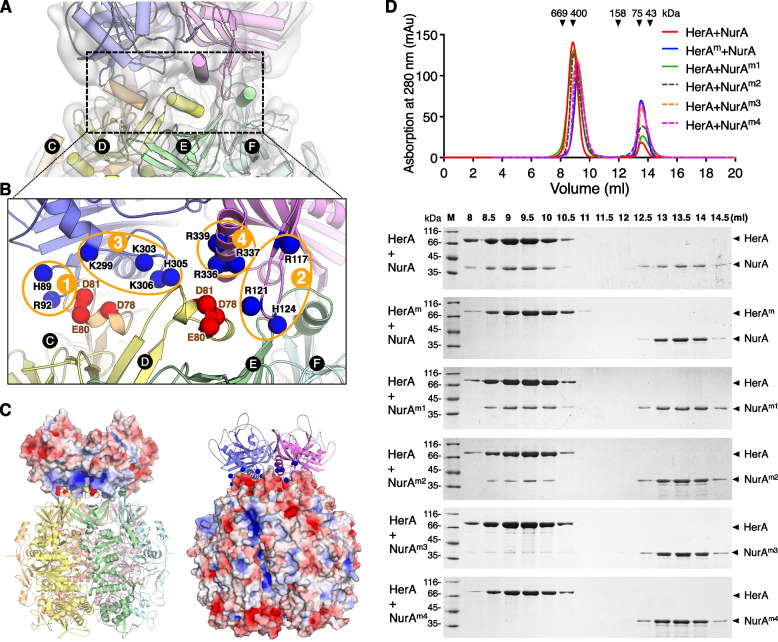
Analysis of the drNurA-HerA interaction interface. **A** The protein interaction interface between drNurA and the HAS-barrel domains of drHerA. The subunits of drHerA and drNurA are labeled and highlighted in distinct colors. The surface around the drNurA-HerA interface is extracted from the real density of the cryo-EM data obtained in this study. **B** The zoom in view of the drNurA-HerA interaction interface. Different protomers are colored differently. The carbon atoms on the Cα backbone of residues which might be essential for interactions, are shown as spheres and colored according to their electrostatic potential (basic residues are colored blue and acidic residues are colored red). **C** Electrostatic properties of drNurA and drHerA. The electrostatic potentials of drNurA and drHerA were calculated separately with APBS and mapped onto the solvent-accessible surface of the structure at contouring levels of ±5 kT (blue/red). **D** Gel filtration analysis of the drNurA-HerA complex formation. The drNurA-HerA mixture was analyzed with Superdex S200 HR 10/300 column. Input drHerA and drNurA were in a molar ratio of 2:1. Peaks of each injection were reconstructed using GraphPad Prism software. Fractions eluted were resolved by 12% SDS-PAGE. HerA^m^, HerA with point mutations D78A, E80A, and D81A; NurA^m1^, NurA with point mutations H89A and R92A; NurA^m2^, NurA with point mutations R117A, R121A, and H124A; NurA^m3^, NurA with point mutations K299A, K303A, H305A, and K306A; NurA^m4^, NurA with point mutations R336A, R337A, and R339A

The same as archaeal NurA-HerA, physical interactions between drHerA and drNurA are essential for their interdependent activities [22]. We analyzed the electrostatic properties of drNurA and drHerA separately (Fig. 3C). The interaction interface of drHerA was found to be highly negatively charged and this charge is mainly mediated by three conserved acidic amino acid residues on drHerA (Asp78, Glu80, and Asp81) (Fig. 3B). Analytical gel filtration chromatography indicated that replacement of these three acidic residues with alanine at the same time resulted in a complete loss of drHerA-drNurA interaction (Fig. 3D). On the other hand, the interaction interface of drNurA is highly positively charged (Fig. 3C). Four putative interaction motifs on drNurA were specified, with each containing multiple conserved basic amino acid residues (Fig. 3B). They are motif-1 (the residues between β2 and β3), motif-2 (the residues between β3 and α1), motif-3, (the residues between α3 and α4), and motif-4 (the residues between α4 and α5). Site-directed mutagenesis of these basic amino acid residues on motif-3 (Lys299, Lys303, His305, and Lys306) and motif-4 (Arg336, Arg337, and Arg339) significantly weakened its interaction with drHerA (Fig. 3D). Site-directed mutagenesis of these basic amino acid residues on motif-1 (His89 and Arg92) and motif-2 (Arg117, Arg121, and His124) slightly weakened the interaction. Therefore, the interaction of drNurA-HerA is mainly mediated by ionic interactions rather than hydrophobic interactions, which is different from that of archaeal NurA-HerA [6, 7, 13]. The motifs for NurA-HerA interaction appeared to be evolutionarily conserved in *Deinococcus* genus (Additional file 4: Fig. S4; Additional file 5: Fig. S5). Despite the related amino acids cannot be well aligned in other bacteria, there are numerous acid/basic residues nearby (especially those acid residues on HerA motif and basic residues on NurA motif-1 and motif-3) (Additional file 4: Fig. S4; Additional file 5: Fig. S5), which result in the negatively charged interaction interface of HerA and the positively charged interaction interface of NurA, and therefore imply a common interaction model for bacterial NurA-HerA.

Furthermore, we noticed that from the overall structure interface of drNurA-HerA that each NurA protomer forms contact with each of the four adjacent HAS domain regions, despite that drHerA and drNurA form a complex at a molar ratio of 6:2. As shown in Fig. 3A, B, subunits A and D of drHerA interact with both NurA monomers. Subunits D, E, F, and A of drHerA form contacts with the same NurA protomer, while subunits A–D of drHerA form contacts with the other NurA protomer. Such organization indicates a tight coupling of drNurA and drHerA and might be essential for the regulation of their interdependent activities.

### DNA and drNurA enhanced the ATPase activity of drHerA

When using magnesium as a catalytic metal, drHerA showed poor ATPase activity, 10- to 100-fold lower than that of ssoHerA, even when DNA and drNurA were present [13]. In this research, the replacement of magnesium with manganese enhanced the ATPase activity of drHerA (Table 2). In contrast, a mutation in the Walker-B motif made drHerA ATPase inactive (Table 2, drHerA^E487A/E488A^). Although drHerA alone displayed weak ATPase activity, the addition of ssDNA or dsDNA significantly increased the ATPase activity, indicating that drHerA is a DNA-dependent ATPase. DrNurA alone did not show any ATPase activity. The presence of drNurA further enhanced the ATPase activity of drHerA, especially in the presence of dsDNA, when the ATPase activity of the drNurA-drHerA complex was 70-fold higher than that of drHerA. On the other hand, in the presence of ssDNA, the ATPase activity of the drNurA-HerA complex was 10-fold higher than that of drHerA. Although both ssDNA and dsDNA can further stimulate the ATPase activity of the drNurA-HerA complex, the enhancement of ssDNA was ~7-fold higher than that of dsDNA. However, the enhancement was modest when drHerA^m^, a mutant that almost completely lacks drNurA binding ability, was used instead (Table 2). Moreover, when drHerA^m^ was used instead of drHerA, the ATPase activities of the drNurA-HerA^m^ complex in the presence of ssDNA and dsDNA were significantly lower than those of the drNurA-HerA complex in the presence of ssDNA and dsDNA (Table 2).

**Table 2:** ATPase analysis of drNurA-HerA and mutant

HerA^b^	NurA^b^	ATPase mol/protein/min (no DNA)	ATPase mol/protein/min (ss DNA)	ATPase mol/protein/min (ds DNA)
wt^a^	-	<<0.01	<<0.01	<<0.01
-	wt^a^	<<0.01	<<0.01	<<0.01
wt^a^	wt^a^	0.08±0.03	0.52±0.09	0.45±0.07
wt	wt	0.42±0.23	28.63±1.78	4.18±0.36
wt	-	<<0.01	1.69±0.09	0.09±0.04
-	wt	<<0.01	<<0.01	<<0.01
HerA^m^	wt	<<0.01	6.01±1.54	0.38±0.09
487/8A	wt	<<0.01	<<0.01	<<0.01
495A	wt	0.46±0.15	6.53±1.11	0.72±0.14
552A	wt	0.39±0.21	9.68±1.81	0.86±0.19
96A	wt	0.45±0.17	27.93±1.99	4.01±0.46
wt	53A	0.43±0.09	24.23±2.38	3.04±0.45
wt	201A	0.46±0.13	21.11±1.98	3.81±0.39
wt	309A	0.34±0.12	28.31±2.71	2.98±0.51
wt	dPin	0.27±0.02	15.85±2.04	0.10±0.04
wt	8A	0.36±0.16	26.64±1.76	0.28±0.08
wt	13A	0.39±0.11	27.13±1.01	3.94±0.19

^a^indicate Mg^2+^ was used instead of Mn^2+^ in this assay

^b^ indicate the addition of wild type or mutated proteins

The measurements were repeated more than six times

### ATP-independent and drHerA-independent nicking endonuclease activity of drNurA against the ssDNA loop

**Fig. 4.**
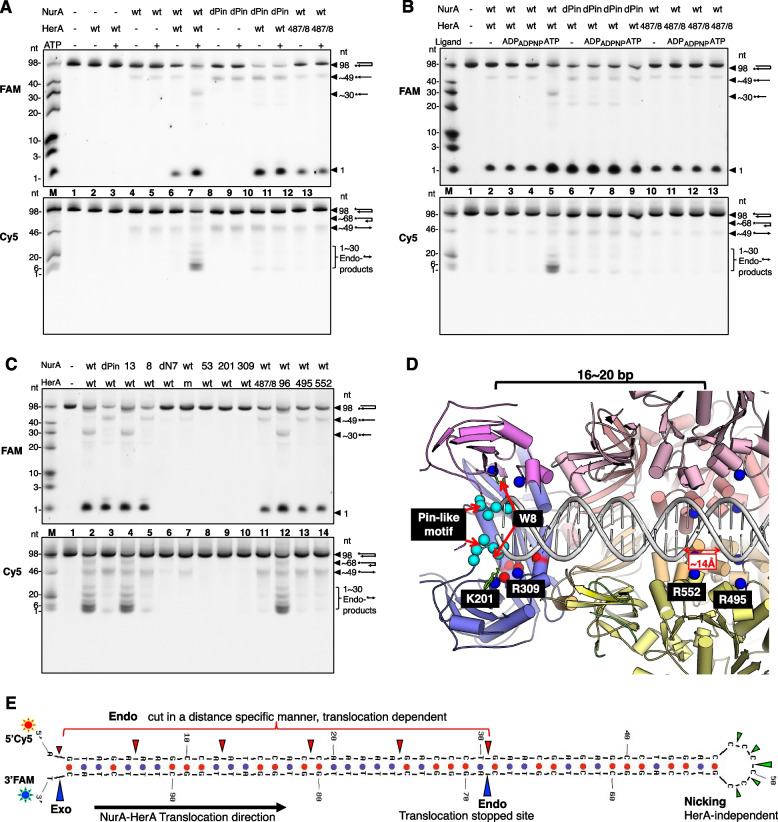
The DNA end resection activities of drNurA-HerA and mutants. **A–C** The DNA end resection activities of different mutants have been compared on the same gel. Briefly, 400 nM substrate DNA was incubated with drNurA (4 μM protomer) and drHerA (12 μM protomer) in the presence of 2 mM MgCl_2_ and 8 mM MnCl_2_. Whenever needed, 1 mM ligand (ADP, ADPNP, or ATP) was added to the reaction system. Products were analyzed on 15% denaturing TBE-PAGE. The same gels were imaged at FAM fluorescent mode (upper) and Cy5 fluorescent mode (lower) separately. The types of the generated products before denaturing have been annotated at the right side of the gel to aid the interpretation of the bands on the gels. Markers were created by mixing different lengths of 3′ FAM or 5′ Cy5 labeled DNA oligos together. dPin, NurA^dPin^; 487/8, HerA^E487A/E488A^; m, HerA^m^; 13, NurA^F13A^; 8, NurA^W8A^; dN7, drNurAdN7; 53, NurA^D53A^; 201, NurA^K201A^; 309, NurA^R309A^; 96, HerA^R96A^; 495, HerA^R495A^; 552, HerA^R552A^. **D** The predicted drNurA-HerA-dsDNA complex model. dsDNA was docked into the DNA binding channel of drHerA. The carbon atoms on the Cα backbone of the residues which were predicted important for translocation and catalysis are shown as spheres and are colored blue and red, respectively. The pin-like motif is shown as spheres and colored cyan. **E** The cutting pattern analysis of the hairpin substrate O1. Putative major cutting sites are marked by arrows. Red arrows indicate that the cleavage requires drNurA-HerA complex in a translocation mode (during translocation), the blue arrows indicate that the cleavage requires drNurA-HerA complex in a steady state mode (translocation stopped or initiated), and the green arrows indicate that the cleavage are drHerA independent and ATP independent. The size of the arrow indicates the digestion efficiency of each site, that the bigger the arrow, the more efficient the cutting

**Fig. 5.**
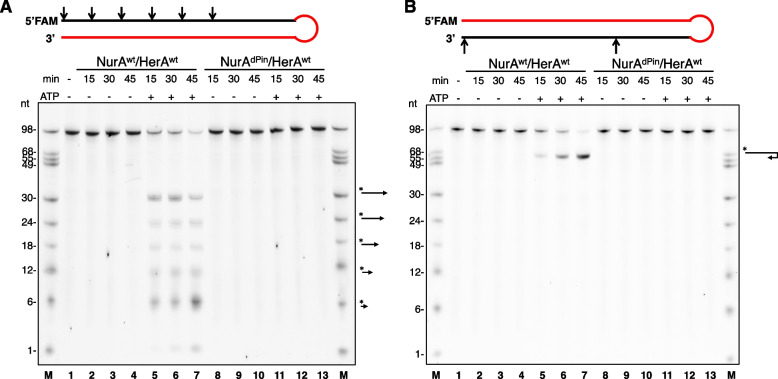
End resection of each DNA tail by drNurA-HerA. **A** The 5′ end resection activity of drNurA-HerA has been analyzed using substrate O3. **B** The 3′ end resection activity of drNurA-HerA has been analyzed using substrate O4. 400 nM 5′ FAM-labeled substrate DNA (O3 or O4) was incubated with drNurA (4 μM protomer) and drHerA (12 μM protomer) in the presence of 2 mM MgCl_2_ and 8 mM MnCl_2_. Reactions were carried out in the absence or in the presence of 1 mM ATP, incubated at 37°C for 15, 30, or 45 min. Reactions were stopped by stop buffer, followed by boiling at 100°C for 5 min and flash-cooling on ice for 10 min. Products were analyzed on 15% denaturing TBE-PAGE and gels were imaged at FAM fluorescent mode. The types of generated products before denaturing have been annotated at the right side of the gel to aid the interpretation of the bands on the gels. Markers were created by mixing different lengths of 5′ FAM-labeled DNA oligos together. The cutting patterns of each hairpin substrate were shown at the top of each gel. Red area indicates these bases were linked by phosphorothioate bonds, which can protect this area of strands from digesting by nuclease. Arrows indicate putative cleavage sites in the presence of ATP

HerA and NurA not only cooperatively decompose ATP but also cooperatively process the 5′ and 3′ tails of dsDNA. HerA mediates DNA translocation, and NurA digests the DNA substrate. To monitor the cutting patterns of both tails of dsDNA at the same time, we synthesized 98-nt DNA (O1, Additional file 6: Table S1). The 3′ end of the DNA was labeled with FAM, and the 5′ end of the DNA was labeled with Cy5. The DNA self-annealed into a hairpin structure with a 46-bp duplex and a 6-nt single-stranded loop (Fig. 4E), preventing the drNurA-HerA complex from binding to it from its loop end. The duplex end rather than the loop end of the hairpin-structured DNA was able to thread through the pore of the complex. The same gels were imaged in FAM fluorescent mode and Cy5 fluorescent mode separately. Because the fluorophore affects the migration rate, bands with the same length but with different labels might migrate to different positions on the same gel. Moreover, we synthesized the 98-nt DNA oligos O3 and O4 (Additional file 6: Table S1). The 5′ ends of O3 and O4 were labeled with FAM, and the sequences of O3 and O4 were the same as that of O1. The bases on the 3′ tail of O3 (bases 46–98) and the bases on the 5′ tail of O4 (bases 1–53) were linked by phosphorothioate bonds (Fig. 5A, B). The phosphorothioate bonds rendered the internucleotide linkages resistant to nucleolytic degradation. Therefore, O3 was used to monitor the resection of the 5′ tail without any interference from the resection of the 3′ tail, and O4 was used to monitor the resection of the 3′ tail without any interference from the resection of the 5′ tail.

In the absence of ATP and drHerA, the nuclease activity of drNurA against O1 was detected. The 3′ end of O1 was labeled with FAM, and the 5′ end of the DNA was labeled with Cy5. After the incubation of O1 with drNurA, a FAM-labeled band with an approximate size of 49 nucleotides (nt) and a Cy5-labeled band with an approximate size of 49 nt appeared on TBE gels (Fig. 4A, lane 4, both the upper and lower gels), suggesting that drNurA nicks the single-stranded loop of O1. Using a gel with a higher resolution and a marker with higher precision, we confirmed that the sizes of the nucleolytic products were in the range of 47–51 nt (Additional file 7: Fig. S6), indicating that drNurA may nick the 6-nt single-stranded loop at any location along the loop (Fig. 4E). Moreover, the nuclease activity of drNurA was ATP independent and drHerA independent (Fig. 4A, lanes 4 and 5, the upper and lower gels), which is consistent with a previous report on the nicking endonuclease activity of drNurA against closed-circular DNA [22]. Meanwhile, there were no such product bands as for O3 and O4, in which the single-stranded loop areas of the hairpin substrates were modified by phosphorothioate bonds to protect them from digestion (Fig. 5A, B).

### DNA translocation-dependent generation of a recombinogenic 3′ ssDNA tail by the drNurA-HerA complex

We further analyzed the patterns of the cutting of both tails of the blunt-ended and hairpin-structured DNA by the drNurA-HerA complex. To interpret the cutting process, a model for a drNurA-HerA-dsDNA complex was constructed, and the cutting sites on the DNA substrate were predicted. The model and cutting sites are shown in Fig. 4 D and E, respectively.

In the presence of ATP, the drNurA-HerA complex showed endonuclease activities against the 5′ tail of O1 (Fig. 4A, lane 7, the lower gel) and the 5′ tail of O3 (Fig. 5A, lanes 5–7), producing oligonucleotides rather than mononucleotides (1-nt nucleolytic products). Endonucleolytic cutting occurred every 5–6 bases on the 5′ tail of O3 (Fig. 5A, lanes 5–7). The digestion of the 5′ tail was blocked when ATP was absent or replaced by an ATP-hydrolysis product (ADP) or a nonhydrolytic ATP analog (ADPNP) (Fig. 4B, lanes 2–5, the lower gel). Moreover, no digestion was detected when ATPase-inactive drHerA (drHerA^E487A/E488A^, Fig. 4B, lanes 10–13, the lower gel) or putatively translocation-defective drHerA (drHerA^R495A^ or drHerA^R552A^; Fig. 4B, lanes 10–13, the lower gel; Fig. 4C, lanes 11, 13, and 14, the lower gel) was used.

Regardless of the absence or presence of ATP, ADP, or ADPNP, the digestion of the 3′ tail of the hairpin-structured DNA by the drNurA-HerA complex generated FAM-labeled bands of 1-nt nucleolytic products (Fig. 4A, lanes 6 and 7, the upper gel; Fig. 4B, lanes 2–5, the upper gel). The exonuclease activity of the complex was also detected when ATPase-inactive drHerA (drHerA^E487A/E488A^, Fig. 4B, lanes 10–13, the upper gel) or putatively translocation-defective drHerA (drHerA^R495A^ or drHerA^R552A^; Fig. 4C; lanes 11, 13, and 14; the upper gel) was used instead of wild-type drHerA. The exonuclease activity occurs only at the edge of the 3′ tail because exonucleolytic-product ladders were not observed when O4 was used as a substrate, even when ATP was present (Fig. 5B, lanes 5–7). FAM-labeled ladders of exonucleolytic products will appear if the drNurA-HerA complex keeps digesting the 3′ tail of the hairpin-structured DNA exonucleolytically during translocation. We speculate that the ATP-independent exonuclease activity of the drNurA-HerA complex against the 3′ tail of the duplex DNA occurs due to a “breathing” effect; that is, the first few bases of the blunt-ended dsDNA can be melted spontaneously. In the presence of ATP, endonucleolytic products were generated. A 30-nt band appeared after digestion of the 3′ tail of O1 (Fig. 4A, lane 7, the upper gel), and 68-nt bands appeared after digestion of the 3′ tail of O4 (Fig. 5B, lanes 5–7). DNA translocation would stop when the length of DNA became too short to bind the translocation motif of drHerA. In the drNurA-HerA-dsDNA model (Fig. 4D), the distance between the catalytic core of drNurA and putative translocation motif of drHerA was approximately 16 bp. The distance is consistent with the remaining length of the 46-bp duplex DNA after the digestion of 30 nt in each tail of the duplex DNA by the drNurA-HerA complex (Fig. 4E). The endonucleolytic-product bands (the 30-nt band for O1 and 68-nt bands for O4) may appear because the drNurA-HerA complex barely digests the 3′ tail of the DNA duplex during translocation but applies strong endonuclease activity against the 3′ tail when translocation stops.

To check whether His-tags would affect the activities of drNurA and drHerA, we also produced proteins without His-tags according to a previous reference [22] and compared the nuclease activities between the His-tagged drNurA-HerA and drNurA-HerA without tag. His-tags did not influence the activities of drNurA-HerA (Additional file 8: Fig. S7). Therefore, we expected the His-tags not to affect the activities of drNurA-HerA in this study.

In summary, DNA end resection is highly regulated by the translocation states of drNurA-HerA. The digestion mainly occurred on the 5′ tail of a duplex DNA during translocation, but it occurred mainly on the 3′ tail of a duplex DNA when translocation was stopped (or just initiated), revealing a mechanism for the DNA translocation-dependent generation of a recombinogenic 3′ ssDNA tail by the drNurA-HerA complex.

### A pin-like motif is essential for dsDNA unwinding during DNA end resection

While the pore size of the drHerA hexamer is large enough to allow the passage of dsDNA, the pore formed by the NurA dimer is too narrow for the passage of DNA. Therefore, duplex DNA must be unwound to allow the passage of each of its strands into the catalytic sites of NurA. A pin-like motif protruded from each drNurA monomer and split the narrow pore formed by the NurA dimer into two channels that only allowed the passage of ssDNA (Fig 2C; Fig. 4D). Endonucleolytic cutting was not observed on either the 3′ tail or the 5′ tail when the drNurA^dPin^-HerA complex was used (Fig. 4A, lanes 10–11; Fig. 4B, lanes 6–9; Fig. 4C, lane 3; Fig. 5A, B, lanes 8–13). DrNurA^dPin^ is a drNurA mutant in which the pin-like motif (amino acid residues 10–15) was deleted. The deletion of the motif impairs the DNA unwinding ability of the drNurA-HerA complex rather than disrupting the catalytic core of drNurA because the drNurA^dPin^-HerA complex, similar to the drNurA-HerA complex, was able to introduce endonucleolytic nicks in the single-stranded loop of the hairpin-structured DNA and digest the base at the edge of the 3′ tail of the DNA in an ATP-independent manner (Fig. 4A, lanes 8–11; Fig. 4B, lanes 6–9; Fig. 4C, lane 3).

Usually, there will be an aromatic residue on the pin-like motif to form a stacking interaction with the DNA base and aid the splitting of the DNA duplex. However, the replacement of a conserved phenylalanine residue on the drNurA pin-like motif with an alanine residue, which resulted in the mutant drNurA^F13A^, did not change the DNA-end-resection efficiency of the complex (Fig. 4C, lane 4). In contrast, the replacement of a conserved tryptophan residue located near the pin-like motif with an alanine residue, which generated a drNurA^W8A^ mutant, reduced the DNA end resection efficiency of the drNurA^W8A^-HerA complex (Fig. 4C, lane 5) compared to that of the drNurA^dPin^-drHerA complex (Fig. 4C, lane 3). In the presence of dsDNA, the ATPase activities of the drNurA^dPin^-drHerA and drNurA^W8A^-HerA complexes were 40-fold and 15-fold lower than that of the drNurA-HerA complex, respectively (Table 2). In the presence of ssDNA, the ATPase activities of the drNurA^dPin^-HerA and drNurA^W8^-drHerA complexes were 1.8-fold and 1.1-fold lower than that of the drNurA-HerA complex, respectively. Mutations in drNurA may impede dsDNA unwinding and dsDNA translocation, but they have less influence on ssDNA translocation.

### Key residues responsible for DNA digestion on drNurA

Site-directed mutagenesis was performed on several conserved basic residues that may aid DNA threading or catalysis on drNurA. The DNA end resection activities of different drNurA proteins in complex with wild-type drHerA were compared. It is worth noting that drNurA^K201A^-HerA and drNurA^R309A^-HerA, similar to the previously confirmed catalytic site mutant drNurA^D53A^-HerA complex [22], completely lost their nuclease activity, including their ability to digest the base at the edge of the 3′ tail in the absence of ATP (Fig. 4C, lanes 8–10). This indicates that residues K201 and R309 on drNurA assist in catalysis. Indeed, the corresponding residue of K201 on ssoNurA (K202) was suggested to interact with phosphate groups flanking scissile phosphodiester bonds [13]. On that basis, K201 and R309 may interact with phosphate groups, and W8, which was located very close to K201 and R309 (Fig. 4D), may form a π-stacking interaction with the DNA base, positioning the nucleotide correctly for DNA digestion. The ATPase activities of the drNurA^D53A^-HerA, drNurA^K201A^-HerA, and drNurA^R309A^-HerA complexes were not significantly different from that of the drNurA-HerA complex (Table 2).

### Key residues responsible for DNA translocation on drHerA

In a study of the *Pseudomonas aeruginosa* FtsK-dsDNA complex, basic residues protruding into the pore from two loops (loops on β4-β5 and β5-β5b at the TR motif) of each FtsK subunit were found to interact with the phosphodiester backbone on both strands of the minor groove and to mediate translocation (PDB ID: 6T8O) [28]. We docked dsDNA into the drNurA-HerA channel and found two conserved basic residues (R495 [located between β3 and β4] and R552 [located between β5 and β5b at the TR motif]), protruding into the pore that might assist in translocation (Fig. 1D; Fig. 4D). R495 and R552 are located at different motifs of each drHerA subunit, and the corresponding residue of R495 on ssoHerA (K363) (Additional file 9: Fig. S8) has been proven to be essential for DNA translocation [14]. R495 and R552 undergo a vertical arrangement along the dsDNA channel, and the distance between them is ~14 Å (Fig. 4D), which is approximately equal to the width of the groove of dsDNA. Therefore, the two residues are expected to hold both strands of dsDNA and translocate the dsDNA in a similar manner to FtsK. The significance of R495 and R552 in DNA translocation was demonstrated by the complete loss of the 5′-end-resection activities of the drNurA-HerA^R495A^ and drNurA-HerA^R552A^ complexes (Fig. 4C, lanes 13 and 14) and the greatly attenuated DNA-dependent ATPase activities of the complexes (Table 2). Two residues located at the N-terminus of the RecA-like domain (K377 and R380) of FtsK have been found to interact with dsDNA [28]. The role of the conserved arginine residue (R96) in the HAS domain of drHerA may be similar to that of K377 and R380. However, drNurA-HerA^R96A^ did not show any DNA end resection (Fig. 4C, lane 12) or ATPase defects (Table 2).

### Survival rates of D. radiodurans cells after treatment with DNA-damaging agents

To investigate the function of the drNurA-HerA complex in the DNA repair process in vivo, the survival rates of *D. radiodurans* cells after treatment with DNA-damaging agents were analyzed. Compared with the wild-type strain, the double-knockout mutant in which *nurA* and *herA* were knocked out exhibited ~10-fold enhanced resistance to a high dose of far-ultraviolet (FUV, 600 J/m^2^) and a high concentration of mitomycin C (MMC, 40 μg/ml) (Additional file 10: Fig. S9)*.* Complementation of a mutant in which drHerA lost almost all its drNurA binding ability (a drNurA-HerA^m^ mutant), a nuclease-inactive mutant (a drNurA^D53A^-HerA mutant), an ATPase-inactive mutant (a drNurA-HerA^E487A/E488A^ mutant), or a mutant with defective DNA unwinding ability (a drNurA^dPin^-HerA mutant) did not recover the phenotype of the wild-type strain, implying that the residues in which the mutations were performed are critical to the in vivo function of the drNurA-HerA complex (Additional file 10: Fig. S9).

## Discussion

Although numerous structural studies have been carried out on archaeal NurA and HerA, our study provides new structural knowledge on the bacterial homologs. In addition to low sequence identities, structural differences exist, as discussed below, that distinguish bacterial NurA-HerA from archaeal NurA-HerA.

It has been suggested that ATP-free or DNA-bound ssoHerA predominantly exists as heptamers in solution but tends to form hexamers in association with ATP or ssoNurA [10]. The heptameric ssoHerA was revealed to exhibit a greater cavity diameter for dsDNA binding (29 Å) than the hexameric form (20 Å) and hence suggested a potential dsDNA loading mechanism [10]. However, both the crystallographic and EM data of this research indicated that drHerA forms a stable hexamer regardless of the presence or absence of nucleotides (ADP or ADPNP), DNA substrates, or drNurA. The hexameric drHerA harbors a channel with a diameter of ~24.5 Å, which is large enough to accommodate dsDNA. Furthermore, unlike ssoHerA, drHerA undergoes limited interaction with neighboring protomers due to its short C-terminal protrusion and steric hindrance. These structural features indicate that drHerA forms a much looser hexameric ring. Therefore, drHerA may be able to adjust the diameter of its cavity to load dsDNA without inserting or removing any subunit.

Although we failed to crystalize drNurA, the Cα backbone of drNurA built from the low-resolution cryo-EM map and the recently released drNurA-HerA complex structure (PDB ID: 7F6D) from another group [27] imply that the dimerization form of drNurA is different from that of archaeal NurA. There is an extra insertion motif (motif-2) on drNurA that assists its binding with drHerA and that motif is absent in archaeal NurA. A pin-like motif on drNurA, which is also observed in the tmNurA structure (PDB ID: 1ZUP), another bacterial NurA, was found to be important for duplex DNA unwinding. However, the pin-like motif is absent in archaeal NurA. The structural features of archaeal NurA that assist in dsDNA unwinding are still unclear.

Another obvious difference between the drNurA-HerA complex and the archaeal NurA-HerA complex is the interaction mode. Although the interaction between drNurA and drHerA and that between archaeal NurA and HerA shared the same 2:6 stoichiometry, the interaction between drNurA and drHerA is mediated mainly ionic interactions, while the interaction between archaeal NurA and HerA is mediated mainly by hydrophobic interactions [6, 7, 13]. Moreover, each ssoNurA protomer interacts with three adjacent ssoHerA subunits [14]. In contrast, each drNurA protomer interacts with four drHerA subunits, suggesting that the coordination between drNurA and drHerA is stronger or more complicated than that between archaeal NurA and archaeal HerA.

**Fig. 6.**
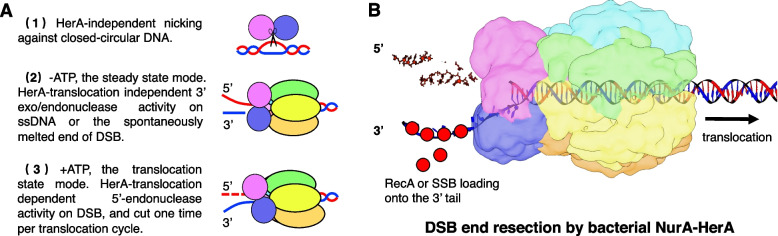
The DNA end resection model of drNurA-HerA. **A** Three different drNurA nuclease activities in the absence/presence of drHerA/ATP are shown. Subunits of drNurA-HerA are highlighted in distinct colors. The 3′ tail and 5′ tail of the duplex DNA are colored blue and red, respectively. The nuclease activities of drNurA are supposed to be regulated in three ways: (1) In the absence of drHerA, drNurA behaves endonuclease nicking against closed-circular DNA substrate. (2) In the presence of drHerA but without translocation (the steady state mode), drNurA prefers introducing an exo/endonuclease cut on the 3′ tail of the duplex DNA. (3) In the presence of drHerA and ATP (the translocation state mode), drNurA prefers digesting the 5′ tail in a translocation-cycle-dependent manner. **B** The DSB end resection model of bacterial NurA-HerA. ATP hydrolysis and translocation of NurA-HerA activate the continuous digestion of the 5′ tail DNA in a translocation-cycle-dependent manner, and create a long recombinogenic 3′ ssDNA end for the subsequent homologous recombination repair

In addition to structural differences, *D. radiodurans* NurA-HerA and archaeal NurA-HerA also exhibit different ATPase activation modes and DNA end resection patterns. For ATPase activity, drNurA alone could stimulate drHerA, and both ssDNA and dsDNA can further stimulate the drNurA-HerA complex. The enhancement of ssDNA was ~7-fold higher than that of dsDNA. However, both ssoHerA and the ssoNurA-HerA complex showed very weak ATPase activity in the absence of DNA [13]. It exhibited the highest ATPase activity on blunt-ended dsDNA substrates, while the stimulatory effect of ssDNA was modest. The addition of ssoNurA could further stimulate ATPase activity only in the presence of blunt-ended dsDNA or dsDNA containing short overhangs [13]. For DNA end resection, drNurA-HerA exhibited translocation-regulated resection, which regularly digested the 5′ tail of blunt-ended dsDNA in an endonucleolytic manner but seldom digested the 3′ tail during translocation. The potential mechanism of such translocation-regulated resection will be discussed further below (Fig. 6). On the other hand, it was reported that both the 5′ tail and 3′ tail of dsDNA would be nucleolysed concurrently by NurA-HerA from *S. solfataricus* and *P. furiosus* [11, 13]. The generation of the recombinogenic 3′ ssDNA tail may depend on the original state of the substrates or the presence of associated proteins such as Mre11-Rad50 [3, 13].

No HerA-DNA or NurA-HerA-DNA complex has yet been reported. Neither a stable drHerA-DNA complex nor a stable drNurA-HerA-DNA complex was obtained in this study. Therefore, it is difficult to accurately describe the regulatory mechanisms at the molecular level. Nevertheless, our data helped us develop reasonable hypotheses about the loading, assembly, translocation, DNA unwinding, and DNA end resection mechanisms of this kind of nuclease-helicase complex, as discussed below.

The first issue is the loading and assembly mechanisms of drNurA and drHerA. The most dominant opinions about the loading methods of ring-like translocases/helicases onto nucleic acid chains include the assembly of a ring around the substrate and the opening of a preformed ring [29, 30]. FtsK, another member of the HerA/FtsK subfamily, is able to assemble from monomers into hexamers around duplex DNA in a process that is independent of ATP or Mg^2+^ cofactors [31]. However, drHerA spontaneously forms a stable hexamer in the absence of DNA or other cofactors. To date, no monomeric form of drHerA has been detected in vitro under any circumstances. No split-ring structure of drHerA has been detected either. The innate stability of drHerA suggests that drHerA binds the broken DNA spontaneously by threading from the broken end so that the drHerA hexameric ring does not have to be disrupted first. In that case, the end of the broken DNA, which usually contains a ssDNA overhang or several base pairs melted simultaneously by a “breathing” effect, can be easily fed into the narrow pore of drNurA.

The second issue is the translocation mechanism of drNurA-HerA. The energy for drNurA-HerA translocation is generated by the regular ATP hydrolysis of drHerA, which acts as the motor protein. Although the translocation mechanism of ring-shaped ssDNA/RNA helicases, such as DnaB, gp4, E1, Rho, and CMG, has been extensively studied [32–36], the mechanism of ring-like dsDNA translocation was provided only by FtsK [28]. FFor a ssDNA/RNA translocase, only one DNA-binding loop of each subunit is observed, which is located between the β3 and β4 strands or the β4 and β5 strands of the RecA-like domain. However, a dsDNA translocase, such as HerA and FtsK, requires two loops/motifs to interact with each strand of duplex DNA, consequently evolving a novel motif (in this study, we named this motif the “TR motif”), which is located between the β5 and β5b strands of the RecA-like domain. The study of the FtsK-dsDNA complex revealed that FtsK, similar to other ring-like translocases, adopts a lock-washer or spiral staircase shape when bound to a substrate and translocates in a sequential “hand-over-hand” manner. During the hand-over-hand movement, the six subunits of the FtsK ring hydrolyze ATP sequentially. We believe that drHerA and FtsK share a similar translocation mechanism since they share similar evolutionary conservation. Therefore, during DNA end resection, the drHerA hexamer turns from a flat ring into a spiral staircase shape and shows gradual conformational changes during its translocation cycle.

The third issue is the unwinding and resection of the broken DNA end, which are cooperatively regulated by drNurA and drHerA. As discussed above, the drHerA hexamer might show gradual conformational changes during its translocation cycle. It is highly possible that the drNurA dimer also undergoes gradual conformational changes, since the drNurA dimer sits tightly at the top of the drHerA hexamer, with each monomer contacting four drHerA subunits. Such conformational changes might influence the nuclease activity of the drNurA-HerA complex during the translocation cycle, and strong endonucleolytic cutting may occur only once per cycle (Fig. 6A). We noticed that endonucleolytic cutting occurred approximately every six bases on the 5′ tail of dsDNA in our DNA end resection assays (Fig. 5A). This is also in agreement with the previous conclusions that hexamers usually translocate over 6 or 12 nt/bp per complete translocation cycle [28, 34]. Archaeal NurA-HerA was reported to generate the 3′ ssDNA tail needed for homologous recombination, with an uncharacterized mechanism. Previous studies suggested possibilities that the 3′ tail of the dsDNA exits from the complex before threading into the NurA active sites so that it can be protected from digestion, and the DNA duplex can be unwound at the same time [13, 14]. However, in the study of drNurA-HerA, the 3′ tail of the dsDNA was found to thread into the drNurA pore, as shown by the detection of strong endonucleolytic cutting within the 3′ tail of the dsDNA. Such endonucleolytic cutting on the 3′ tail only occurred when translocation was initiated/stopped, probably due to the conformational change in drNurA when translocation was initiated/stopped. Our data also indicated that the pin-like motif on drNurA is the key element for melting the DNA duplex. It remains unknown how archaeal NurA-HerA complexes unwind the duplex because such pin-like motifs are not conserved in archaeal NurAs.

Overall, after analyzing our structural and biochemical data, we provided a putative mechanism for DNA end resection mediated by the bacterial NurA-HerA complex. When DNA damage occurs, the NurA-HerA complex recognizes and directly binds the broken DNA end. The binding of DNA and ATP shapes the HerA hexamer into a spiral staircase state and simultaneously causes conformational changes in the drNurA dimer. ATP hydrolysis drives the translocation of NurA-HerA along the DNA duplex, causing continuous and translocation-dependent digestion of the 5′ tail of the dsDNA and the generation of a long 3′ ssDNA for subsequent homologous recombination repair (Fig. 6B). Notwithstanding, additional structural and functional studies are required to elucidate the precise mechanisms of the DNA translocation and DNA end processing that are mediated by the drNurA-HerA complex.

We remained uncertain of the biological role of bacterial NurA-HerA. Although drNurA contains an RNaseH-like domain, no RNaseH activity was detected, even in the presence of drHerA (Additional file 11: Fig. S10). In archaea, NurA-HerA rescues cells from DNA-damaging agents by resecting DSB ends in the initiation process of homologous recombination repair [3–7]. Therefore, the expression of NurA and HerA in bacteria was expected to enhance DNA damage resistance as well. However, we found that the deletion of *nurA* and *herA* genes in *D. radiodurans* improved the tolerance of the bacterium to a high concentration of MMC and a high dose of FUV*.* MMC treatment and high-dose FUV radiation are known to block DNA replication and generate DSBs. The negative effects of the NurA-HerA complex on DNA repair have also been observed in *T. thermophilus* [23, 24]. Therefore, we hypothesized that the bacterial NurA-HerA complex attenuates DSB repair efficiency in vivo.

Above, we suggested a possible model to explain how drNurA-HerA processes DSB end resection and generates a 3′ ssDNA tail in a translocation-regulated manner. However, our biochemical data indicated that the in vitro DSB end resection activity of drNurA-HerA is very weak compared with that of archaeal NurA-HerA (protein concentrations for digestion assays are micromolar compared to nanomolar) [3–7]. Additionally, as observed in our biochemical data, obstruction of the translocation would redirect drNurA-HerA from digesting the 5′ tail to digesting the 3′ tail, generating a nonpreferred substrate for RecA-mediated recombinational repair. Therefore, the drNurA-HerA complex may not be an efficient DSB end resection complex for homologous recombination repair in vivo. In contrast, RecJ-RecQ from *D. radiodurans* (drRecJ-RecQ) exhibits much more efficient DSB end resection than *Escherichia coli* RecJ-RecQ [16]. The existence of the ESDSA pathway in *D. radiodurans* further certifies the importance of drRecJ, which might provide an evolutionary advantage in highly radioresistant bacteria [19–21]. Similar to other bacteria in the *Deinococcus-Thermus* phylum, *D. radiodurans* encodes a stand-alone RecD protein (drRecD) but naturally lacks RecBC. Unexpectedly, the overproduction of RecBC from *E. coli* (ecRecBC) in *D. radiodurans* attenuated its resistance to gamma radiation and FUV radiation [37]. The authors suggested that ecRecBC fails to form a functional complex with drRecD. Moreover, ecRecBC has negative effects on DSB end resection mediated by the drRecJ-RecQ complex due to its strong DSB end binding activity and strong helicase activity but weak DNA end resection activity [37]*.* We speculate that the drNurA-HerA complex competes with the robust drRecJ-RecQ complex in a similar manner to ecRecBC, which results in attenuated DNA repair efficiency in *D. radiodurans*.

## Conclusions

The DNA replication and DNA repair systems are quite different between archaea and bacteria. Actually, bacteria are more accustomed to adopting RecBCD or RecJ-RecQ for DSB DNA end resection. Bacterial NurA-HerA are only present in a few bacteria, such as those from the *Deinococcus-Thermus* phylum, which naturally lacks RecBCD but possess robust RecJ-RecQ DNA end resection system. Here, structural differences were observed between the bacterial and archaeal NurA-HerA complex, which imply they possibly share different molecular mechanisms. For example, the stable hexameric form and a larger DNA binding channel of drHerA indicate a possible different DNA loading mechanism from that of archaeal HerA; an extra pin-like motif on drNurA indicates a possible different DNA unwinding mechanism from that of archaeal NurA; the different interaction mode of drNurA-HerA indicates a possible different cooperation mechanism from that of archaeal NurA-HerA. Our biochemical data partially confirmed the above speculation. In summary, our work provides new insights into the mechanism underlying bacterial NurA-HerA-mediated DSB DNA end resection, and the way this complex digests the 5′ tail of a DNA duplex and provides long 3′ free end for strand invasion in the bacterial homologous recombination process. Our data indicates the bacterial and archaeal NurA-HerA share possibly distinct molecular mechanisms and biological functions.

## Methods

### Construction of the expression vectors

The full-length genes encoding drHerA (residues 1−618 aa) and drNurA (residues 1−349 aa) were amplified from *D. radiodurans* genomic DNA by polymerase chain reactions and cloned into pET28a and pET22b expression vectors, respectively. The former encodes a fused N-terminal 6× His tag, while the latter encodes a fused C-terminal 6× His tag, near the target genes. Site-directed mutagenesis was performed with a QuikChangeTM Site-Directed Mutagenesis Kit from Stratagene (La Jolla, CA), as described previously [16]. The pin-like motif deleted mutant (residues 10–15 aa) was obtained using the same method as site-directed mutagenesis. The fidelity of the mutants was confirmed by sequencing. All successfully constructed expression vectors were transformed into *E. coli* Rossetta (DE3) strain (TransGen Biotech, Beijing). Primers were purchased from Generay Biotech (Shanghai, China). A list of the primers used for the cloning and mutagenesis is provided in Additional file 6: Table S1.

### Expression and purification of the proteins

All proteins were expressed in *E. coli* Rossetta (DE3) cells at 18°C for 18 h, with induction by 0.2 mM isopropyl β-D-thiogalactoside (IPTG).

The proteins drHerA and drNurA were purified in a similar way. In brief, cells were re-suspended in buffer A (20 mM Tris [pH 8.0], 150 mM NaCl, 0.5 mM Tris-[2-carboxyethyl]-phosphine [TCEP], and 5 mM imidazole), lysed by high-pressure cracker and centrifuged at 20,000×*g* and 4°C for 60 min. The supernatant was loaded onto the HisTrap HP column (GE Healthcare, Fairfield, CT), equilibrated with buffer A, washed with 30 mM imidazole, and eluted with 200 mM imidazole. The eluted fractions were subsequently loaded onto the Capto HiRes Q 5/50 column (Cytiva), equilibrated with buffer B (20 mM Tris [pH 8.0], 150 mM NaCl, and 0.5 mM TCEP), and eluted with a linear gradient from 150 mM to 1 M NaCl. Proteins were finally purified by Superdex 200 Increase 10/300 GL column (GE Healthcare) using buffer C (20 mM Tris [pH 8.0], 100 mM NaCl, and 0.5 mM TCEP). Fractions containing the purified proteins were pooled, concentrated, flash-frozen in liquid nitrogen, and stored at −80°C.

Proteins without His-tags were obtained based on previous reference [22], which were expressed in fusion with His and MBP tag, purified, and followed by tobacco etch virus (TEV) enzyme removal of the tags.

### Crystallization and structure determination

Crystallization trials were performed by the sitting drop vapor diffusion method at 289 K. In brief, the freshly purified protein was concentrated to ~10 mg/ml and centrifuged to remove insoluble fractions before crystallization. After a series of screening tests and optimizations, the best crystals of drHerA alone were obtained in condition of 0.1 M HEPES (pH 7.5) and 1.3 M NaAc by the micro-seeding method. Selenium-labeled crystals were also obtained to solve the phase problem. To prepare the drHerA-ADP•AlF_3_ complex, 10 mM MgAc, 5 mM ADP, 20 mM NaF, and 5 mM AlCl_3_ were added sequentially and the mixture was incubated on ice for 10 min before setting up the crystallization. Best crystals were obtained in conditions of 0.2 M MgCl_2_, 0.1 M HEPES (pH 7.8), and 14% PEG 3350. However, the density of ADP, rather than ADP•AlF_3_, was observed in the structure. Cryocooling was achieved by stepwise soaking of the crystals in reservoir solution containing 10, 20, and 30% (w/v) glycerol for 1 min and flash freezing in liquid nitrogen. X-ray diffraction data were collected on beamline BL02U1 at Shanghai Synchrotron Radiation Facility (Shanghai, China) and integrated and scaled with the XDS suite [38]. The drHerA apo structure was determined by single-wavelength anomalous diffraction (SAD) phasing using selenomethionine-substituted protein crystals and refined with the native data. The drHerA-ADP complex structure was determined by the molecular replacement method using drHerA apo structure as the search model. Structures were refined using PHENIX [39] and interspersed with manual model building using COOT [40]. All residues were in the most favorable and allowed regions of the Ramachandran plot. All structural figures were created by PyMOL. The statistics for the data collection and refinement are listed in Table 1.

### Cryo-EM data collection and processing

Two-microlitre aliquots of the purified drHerA protein or the drNurA-HerA complex were applied to graphene-oxide-covered R2/2 300 mesh Cu holey carbon grids (Quantifoil), blotted for 1 s, and then flash-frozen in liquid ethane cooled by liquid nitrogen with Vitrobot Mark IV (Thermo Fisher). The graphene-oxide-covered grids were prepared as previously described [41]. The grids were subsequently transferred to a 200 kV cryo-transmission electron microscope (Tecnai F20), and images were recorded with a CCD 2K camera (Gatan Orius SC200). A total of 1200 movie stacks of the drNurA-HerA complex dataset were automatically collected using EPU software. For each dataset of HerA alone, HerA with ADP, HerA with ADPNP, and HerA with dsDNA, 100 movie stacks were automatically collected using EPU software.

Movie stacks were aligned and summed using Motioncor2 [42]. Template-free particle picking was done in Gautomatch using a circular diameter of 180 Å. Contrast transfer function parameters were estimated for each micrograph using Gctf [43]. Particle extractions were done and two rounds of two-dimensional (2D) classifications were performed in RELION3 [25]. For the structural analysis of drHerA, only top views were selected for further 2D classification. Six 2D classes were obtained for each dataset. For the structural analysis of the drNurA-HerA complex, all the real particles from 2D classification were selected and further subjected to 3D refinement with C2 symmetry.

The drNurA initial model was predicted by the online protein structure prediction tool AlphaFold2 [25]. Both the drHerA structure solved by crystallography and the predicted drNurA structure were initially fitted into the map of the drNurA-HerA complex by Chimera [26] and then edited by jelly-body refinement with Refmac5 [44] in CCP-EM [45]. The Cα backbone of the predicted drNurA was further manually refined in COOT [40] according to the real density of the EM data, followed by cycles of real-space refinements with PHENIX [46].

### Analytical gel filtration

The dimerization of drNurA and the physical interactions between drHerA and drNurA were investigated using analytical gel filtration as previously described with some modifications [22]. In brief, 300 μl gel filtration buffer (20 mM Tris [pH 8.0], 100 mM NaCl, and 1 mM TCEP) containing drNurA alone (3 mol protomer) or drHerA-NurA mixture (6 mol HerA protomer and 3 mol NurA protomer) were spun at 15,000×*g* for 5 min to remove any precipitated protein. Afterward, the supernatant was then loaded onto a Superdex 200 Increase 10/300 GL column (GE Healthcare). Fractions (0.5 ml) were collected and analyzed by 12% SDS-PAGE. The sizes of calibration proteins at the positions where they eluted were marked on the *x*-axis based on the method described for the gel filtration calibration kit HMW (GE Healthcare).

### Nuclease activity assays

DNA substrates were synthesized by Generay Biotech (Shanghai, China) (Additional file 6: Table S1). The substrates were self-annealed to generate a hairpin structure with a 46-base-pair (bp) duplex and a 6-nt loop, using a gradient cooling program. Nuclease activity assays were performed according to previously reported methods, with some modifications [22, 47]. A typical reaction mixture, if not mentioned specially, contains drNurA (2 μM dimer or 4 μM protomer), drHerA (2 μM hexamer 12 μM protomer), and 400 nM substrate DNA in reaction buffer (25 mM Tris-HCl [pH 7.5], 60 mM KCl, 1 mM DTT, 2 mM MgCl_2_, 8 mM MnCl_2_, and 0.1 mg/ml bovine serum albumin [BSA]). The mixtures were incubated at 37°C for 30 min, and reactions were stopped by adding the same volume of 2× reaction stop buffer (95% formamide, 50 mM EDTA, 0.05 mg/ml protease K, and 0.01% bromophenol blue), followed by boiling at 100°C for 5 min and flash-cooling on ice for 10 min. When necessary, 1 mM ligand (ADP, ADPNP, or ATP) was added to the reaction system. The reaction products were analyzed on 15% denaturing polyacrylamide gels containing 7 M urea in Tris-borate-EDTA buffer. Gels were imaged by fluorescence mode (FAM or Cy5) on ChemiScope6100 (Clinx Science Instruments, Shanghai).

### ATPase activity

The ATPase activity of NurA-HerA was measured using a coupled assay that monitors ADP release as described previously [48]. A final concentration of 100 μM NADH, 0.5 mM phosphoenolpyruvate, 100 U/ml pyruvate kinase (Sangon), and 20 U/ml lactate dehydrogenase (Sangon) were used in all reactions. Typical reactions were conducted using 400 nM purified drNurA-HerA or mutants, with or without 4 μM 98 nt ssDNA (98nt_F) or 98 bp dsDNA (annealed by 98nt_F and 98nt_R) and 1 mM ATP in reaction buffer (50 mM Tris [pH 8.0], 100 mM NaCl, 5 mM MnCl_2_, 10 mg/ml bovine serum albumin, and 0.5 mM TCEP). All components, except ATP, were mixed prior to transferring to a Black Opaque 384-well microplate (CORNING) that had been pre-incubated at 37°C. Reactions were initiated with adding of ATP. Reactions were monitored fluorescently using an excitation of 335 nm and an emission of 469 nm at 37°C with the SpectraMax M5E microplate reader (Molecular Devices, USA). The measurements were repeated more than six times. All reaction rates were determined from the initial linear rate, and reaction kinetics were analyzed assuming a Michaelis–Menten model by GraphPad Prism 9 (San Diego, USA).

### DNA-damaging agent survival rate assays

The *nurA*/*herA* deletion strain and the related complementation strains were generated as previously described [22, 47, 49]. The *nurA* and *herA* genes were deleted and complemented together, as an operon. Survival rate assays were performed as previously described, with some modifications [22, 47, 49]. Cells were grown in TGY medium with the appropriate antibiotics to early exponential phase (OD_600_= 0.6–0.8). For FUV treatment, cells were first 10-fold serially diluted at each step, and dotted on the TGY plates. After complete absorption, the plates were exposed to xenon lamp (254 nm) at the dose of 600 J/m^2^. For mitomycin C (MMC) treatment, cells were incubated with 40 μg/ml of MMC at 30°C for 20 min, and then 10-fold serially diluted at each step, and dotted on the TGY plates. Plates were cultured for 2−3 days at 30°C.

## Supplementary Information

**Additional file 1: Figure S1.** Analysis of drHerA folding, hexamerization and ligand binding. A and B, Cross section of RecA-like domains, top view and bottom view of drHerA-ADP bound hexamer (A) and apo hexamer (B). Each subunit is labeled and highlighted in distinct colors. The HAS domains in the top view of drHerA have been hidden in order to exhibit the DNA binding residues (six Arg495 are shown as surface with blue color) clearly. The sizes of drHerA-ADP and ring diameters are measured in PyMOL and labeled.

**Additional file 2: Figure S2.** Details of ssoHerA (A) and paFtsK (B) protomer. Upper, the domain arrangements. The HAS/NTD, RecA-like and helix-bundle domains are colored magenta, yellow and marine, respectively. The motif important for DNA translocation are colored lime green. Lower, cartoon view of two protomers. Each domain is colored the same as domain arrangement. The neighboring protomer is colored white. The ATP molecules are shown as sticks.

**Additional file 3: Figure S3.** The superimpositions of the drNurA-HerA complex from this study and from the published data. A, drNurA-HerA model from this study (different subunits are colored differently) and from the published data (colored white, PDB code: 7f6d) were superimposed by PyMOL. Structures are shown as cartoon. B, The zoom-in view of the protein interaction interface between drNurA and the HAS-barrel domains of drHerA. It is the same view as in the Figure 3B, except that the model we built in this study was replaced by the published structure (PDB code: 7f6d). The carbon atoms on the Cα backbone of residues, which might be essential for interaction, are shown as spheres and colored according to their electrostatic potential (basic residues are colored blue and acidic residues are colored red).

**Additional file 4: Figure S4.** Sequence alignments of HerA proteins from different bacteria. The HAS domain, RecA-like domain, helix-bundle domain and TR motif were framed by magenta, yellow, marine and green frames, respectively. The motif for drNurA-HerA interaction was framed with purple frame. dra, *Deinococcus radiodurans*; dmr, *Deinococcus maricopensis*; ddr, *Deinococcus deserti*; dgo, *Deinococcus gobiensis*; dge, *Deinococcus geothermalis*; ttj, *Thermus thermophilus* HB8; tth, *Thermus thermophilus* HB27; tra, *Truepera radiovictrix*; tma, *Thermotoga maritima*. Secondary structural elements are depicted according to the PDB files (dra_HerA, this study), which arrows represent β-sheet, helices represent α-helices and ‘T’s represent ‘turn’s.

**Additional file 5: Figure S5.** Sequence alignments of NurA proteins from different bacteria. The abbreviations of each bacterium are the same as Figure S4. The pin-like motifs were framed with cyan frame. The motifs for drNurA-HerA interaction were framed with orange frames and numbered. Secondary structural elements are depicted according to PDB files (dra_NurA, this study; tma_NurA, PDB ID=1ZUP), which arrows represent β-sheet, helices represent α-helices and ‘T’s represent ‘turn’s.

**Additional file 6: Table S1.** Oligos used in this study.

**Additional file 7: Figure S6.** The cutting pattern analysis of drNurA and drNurA-HerA of the hairpin substrate O1 in the absence of ATP. 400 nM hairpin substrate O1 (with 5′ Cy5 and 3′ FAM labeled) was incubated with 4 μM drNurA alone or drNurA-HerA complex in the presence of 2 mM MgCl_2_ and 8 mM MnCl_2_. Reactions were carried out in the absence of ATP, and incubated at 37°C for 30 min. Reactions were stopped by the stop buffer, followed by boiling at 100°C for 5 min and flash-cooling on ice for 10 min. Products were analyzed on 15% denaturing TBE-PAGE and gels were imaged at FAM fluorescent mode. Markers were created by mixing different lengths of 5′FAM-labeled DNA oligos together.

**Additional file 8: Figure S7.** The DNA end resection activities of His-tagged drNurA-HerA and drNurA-HerA without tag. 400 nM 5′ FAM-labeled substrate DNA (O3 or O4) was incubated with His-tagged drNurA-HerA and drNurA-HerA without tag (2 μM) in the presence of 2 mM MgCl_2_ and 8 mM MnCl_2_. Reactions were carried out in the absence or in the presence of 1 mM ATP, and incubated at 37°C for 30 min. Reactions were stopped by stop buffer, followed by boiling at 100°C for 5 min and flash-cooling on ice for 10 min. Products were analyzed on 15% denaturing TBE-PAGE and gels were imaged at FAM fluorescent mode. The types of the generated products before denaturing have been annotated at the right side of the gel to aid the interpretation of the bands on the gels. Markers were created by mixing different lengths of 5′FAM-labeled DNA oligos together.

**Additional file 9: Figure S8.** Comparisons of the ATP catalytic sites and the DNA translocation related motifs of drHerA (A), ssoHerA (B) and PaFtsK (C). Upper, zoom-in views of the ATP catalytic sites. The ligands, key residues for ATP binding and hydrolysis are shown as sticks. Middle, zoom-in views of DNA translocation related motifs. The key residues for dsDNA translocation are shown as sticks. Lower, the topology diagrams of RecA-like domain. Residues for metal binding, ATP binding, and DNA binding are shown as red, blue, and cyan dots. Insertion domains/motifs are shown as grey triangles.

**Additional file 10: Figure S9.** The phenotypes of drNurA-HerA deletion mutant and complementation strains. Wild type strain, drNurA-HerA deletion mutant (*△*), drNurA-HerA complemented strain (*△+nurA-herA*), drNurA-HerA interaction defect mutant complemented strain (*△+ nurA-herA*^*m*^), the nuclease-inactive mutant complemented strain (*△+nurA*^*D53A*^*-herA*), the ATPase-inactive mutant complemented strain (*△+ nurA-herA*^*E487A/E488A*^), and the unwinding activity defect mutant complemented strain (*△+ nurA*^*dPin*^*-herA*) were treated with different FUV (600 J/m^2^) and MMC (40 μg/ml). Cells were diluted and dotted on plates. Plates were cultured for 2−3 days at 30°C.

**Additional file 11: Figure S10.** The RNaseH activity analysis of drNurA and the drNurA-HerA complex. A 60 nt 5′ FAM-labeled ssRNA or 60 bp RNA/DNA duplex were incubated with 1, 2, or 4 μM drNurA dimer (in the absence or presence of 1, 2, or 4 μM drHerA hexamer), in the presence of 2 mM MgCl_2_ and 8 mM MnCl_2_ at 37°C for 30 min. Reactions were carried out using the same reaction condition as Figure 4A, in the absence or presence of 1 mM ATP. The products were resolved by 15% TBE-urea denaturing gel.

## Data Availability

All data generated or analyzed during this study are included in this published article, its supplementary information files and publicly available repositories. Atomic coordinates and structure factors for the reported crystal structures have been deposited with the Protein Data bank (http://www.wwpdb.org) under accession number 7WRW (DOI: 10.2210/pdb7wrw/pdb) and 7WRX (DOI: 10.2210/pdb7wrx/pdb).

## Abbreviations

dr: *Deinococcus radiodurans*
dsDNA: Double-stranded DNA
ssDNA: Single-stranded DNA
TR: Translocation-related
DSB: Double-stranded break
HR: Homologous recombination
sso: *Sulfolobus solfataricus*
pf: *Pyrococcus furiousus*
tt: *Thermus thermophiles*
tm: *Thermotoga maritima*
EM: Electron microscopy
cryo-EM: Cryo-electron microscopy
ESDSA: Extended synthesis-dependent strand annealing
SAD: Single-wavelength anomalous diffraction
ASCE: Additional strand catalytic ‘E’
HAS: HerA-ATP synthase
IPTG: Isopropyl β-D-thiogalactoside
TCEP: Tris-(2-carboxyethyl)-phosphine
TEV: Tobacco etch virus
FUV: Far-ultraviolet
MMC: Mitomycin C
