# Supplementary material for: Structural and DNA end resection study of the bacterial NurA-HerA complex
Source: BMC Biol. 2023 Feb 24;21:42. doi: 10.1186/s12915-023-01542-0 (PMC9960219; doi:10.1186/s12915-023-01542-0)
Supplement: Supplementary file 1 — Additional file 1: Figure S1. Analysis of drHerA folding, hexamerization and ligand binding. A and B, Cross section of RecA-like domains, top view and bottom view of drHerA-ADP bound hexamer (A) and apo hexamer (B). Each subunit is labeled and highlighted in distinct colors. The HAS domains in the top view of drHerA have been hidden in order to exhibit the DNA binding residues (six Arg495 are shown as surface with blue color) clearly. The sizes of drHerA-ADP and ring diameters are measured in PyMOL and labeled. [file 12915_2023_1542_MOESM1_ESM.pdf]

Additional file 1: Figure S1.

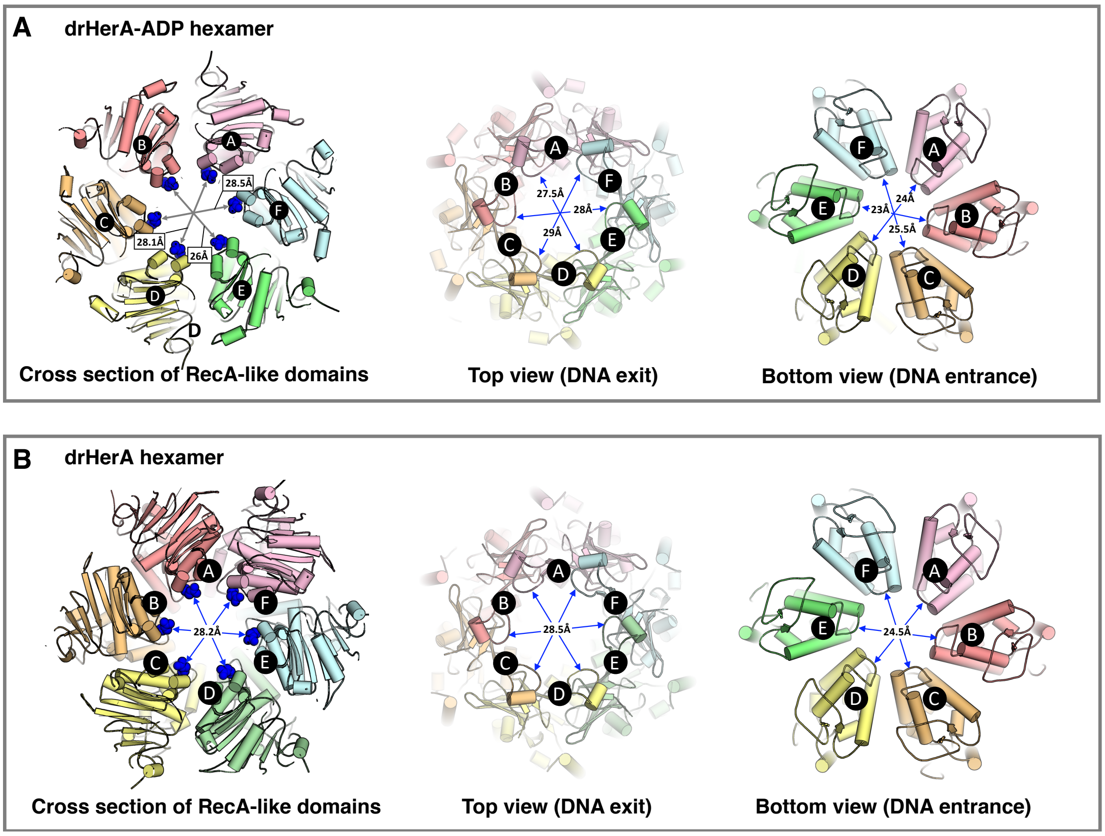

Analysis of drHerA folding, hexamerization and ligand binding.

A and B, Cross section of RecA-like domains, top view and bottom view of drHerA ADP bound hexamer (A) and apo hexamer (B). Each subunit is labeled and highlighted in distinct colors. The HAS domains in the top view of drHerA have been hidden in order to exhibit the DNA binding residues (six Arg495 are shown as surface with blue color) clearly. The sizes of drHerA-ADP and ring diameters are measured in PyMOL and labeled.
