# Supplementary material for: Structural and DNA end resection study of the bacterial NurA-HerA complex
Source: BMC Biol. 2023 Feb 24;21:42. doi: 10.1186/s12915-023-01542-0 (PMC9960219; doi:10.1186/s12915-023-01542-0)
Supplement: Supplementary file 2 — Additional file 2: Figure S2. Details of ssoHerA (A) and paFtsK (B) protomer. Upper, the domain arrangements. The HAS/NTD, RecA-like and helix-bundle domains are colored magenta, yellow and marine, respectively. The motif important for DNA translocation are colored lime green. Lower, cartoon view of two protomers. Each domain is colored the same as domain arrangement. The neighboring protomer is colored white. The ATP molecules are shown as sticks. [file 12915_2023_1542_MOESM2_ESM.pdf]

Additional file 2: Figure S2.

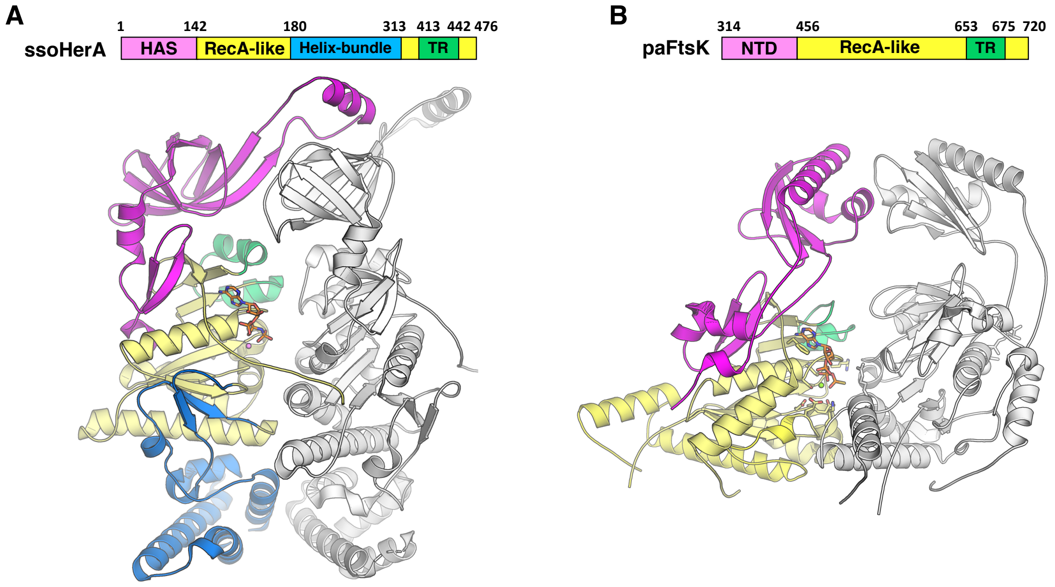

Details of ssoHerA (A) and paFtsK (B) protomer.

Upper, the domain arrangements. The HAS/NTD, RecA-like and helix-bundle domains are colored magenta, yellow and marine, respectively. The motif important for DNA translocation are colored lime green. Lower, cartoon view of two protomers. Each domain is colored the same as domain arrangement. The neighboring protomer is colored white. The ATP molecules are shown as sticks.
