# Supplementary material for: Structural and DNA end resection study of the bacterial NurA-HerA complex
Source: BMC Biol. 2023 Feb 24;21:42. doi: 10.1186/s12915-023-01542-0 (PMC9960219; doi:10.1186/s12915-023-01542-0)
Supplement: Supplementary file 3 — Additional file 3: Figure S3. The superimpositions of the drNurA-HerA complex from this study and from the published data. A, drNurA-HerA model from this study (different subunits are colored differently) and from the published data (colored white, PDB code: 7f6d) were superimposed by PyMOL. Structures are shown as cartoon. B, The zoom-in view of the protein interaction interface between drNurA and the HAS-barrel domains of drHerA. It is the same view as in the Figure 3B, except that the model we built in this study was replaced by the published structure (PDB code: 7f6d). The carbon atoms on the Cα backbone of residues, which might be essential for interaction, are shown as spheres and colored according to their electrostatic potential (basic residues are colored blue and acidic residues are colored red). [file 12915_2023_1542_MOESM3_ESM.pdf]

**Additional file 3: Figure S3.**

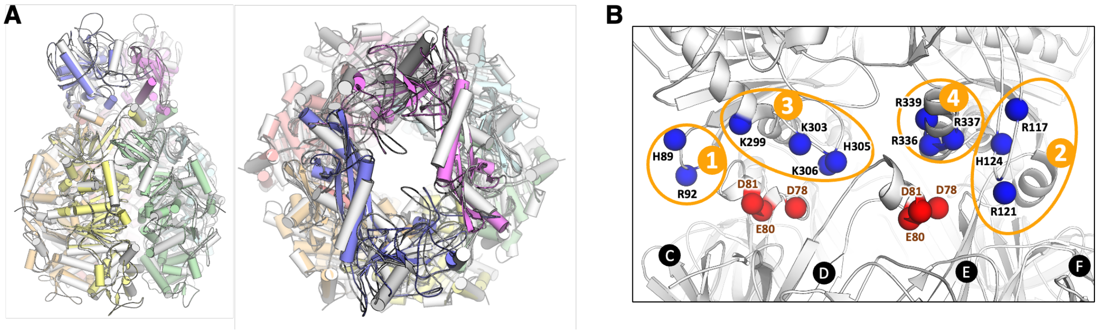


**The superimpositions of the drNurA-HerA complex from this study and from the published data.**

A, drNurA-HerA model from this study (different subunits are colored differently) and from the published data (colored white, PDB code: 7f6d) were superimposed by PyMOL. Structures are shown as cartoon. B, The zoom-in view of the protein interaction interface between drNurA and the HAS-barrel domains of drHerA. It is the same view as in the Figure 3B, except that the model we built in this study was replaced by the published structure (PDB code: 7f6d). The carbon atoms on the Cα-backbone of residues, which might be essential for interaction, are shown as spheres and colored according to their electrostatic potential (basic residues are colored blue and acidic residues are colored red).
