# Supplementary material for: Structural and DNA end resection study of the bacterial NurA-HerA complex
Source: BMC Biol. 2023 Feb 24;21:42. doi: 10.1186/s12915-023-01542-0 (PMC9960219; doi:10.1186/s12915-023-01542-0)
Supplement: Supplementary file 4 — Additional file 4: Figure S4. Sequence alignments of HerA proteins from different bacteria. The HAS domain, RecA-like domain, helix-bundle domain and TR motif were framed by magenta, yellow, marine and green frames, respectively. The motif for drNurA-HerA interaction was framed with purple frame. dra, Deinococcus radiodurans; dmr, Deinococcus maricopensis; ddr, Deinococcus deserti; dgo, Deinococcus gobiensis; dge, Deinococcus geothermalis; ttj, Thermus thermophilus HB8; tth, Thermus thermophilus HB27; tra, Truepera radiovictrix; tma, Thermotoga maritima. Secondary structural elements are depicted according to the PDB files (dra_HerA, this study), which arrows represent β-sheet, helices represent α-helices and ‘T’s represent ‘turn’s. [file 12915_2023_1542_MOESM4_ESM.pdf]

Additional file 4: Figure S4.

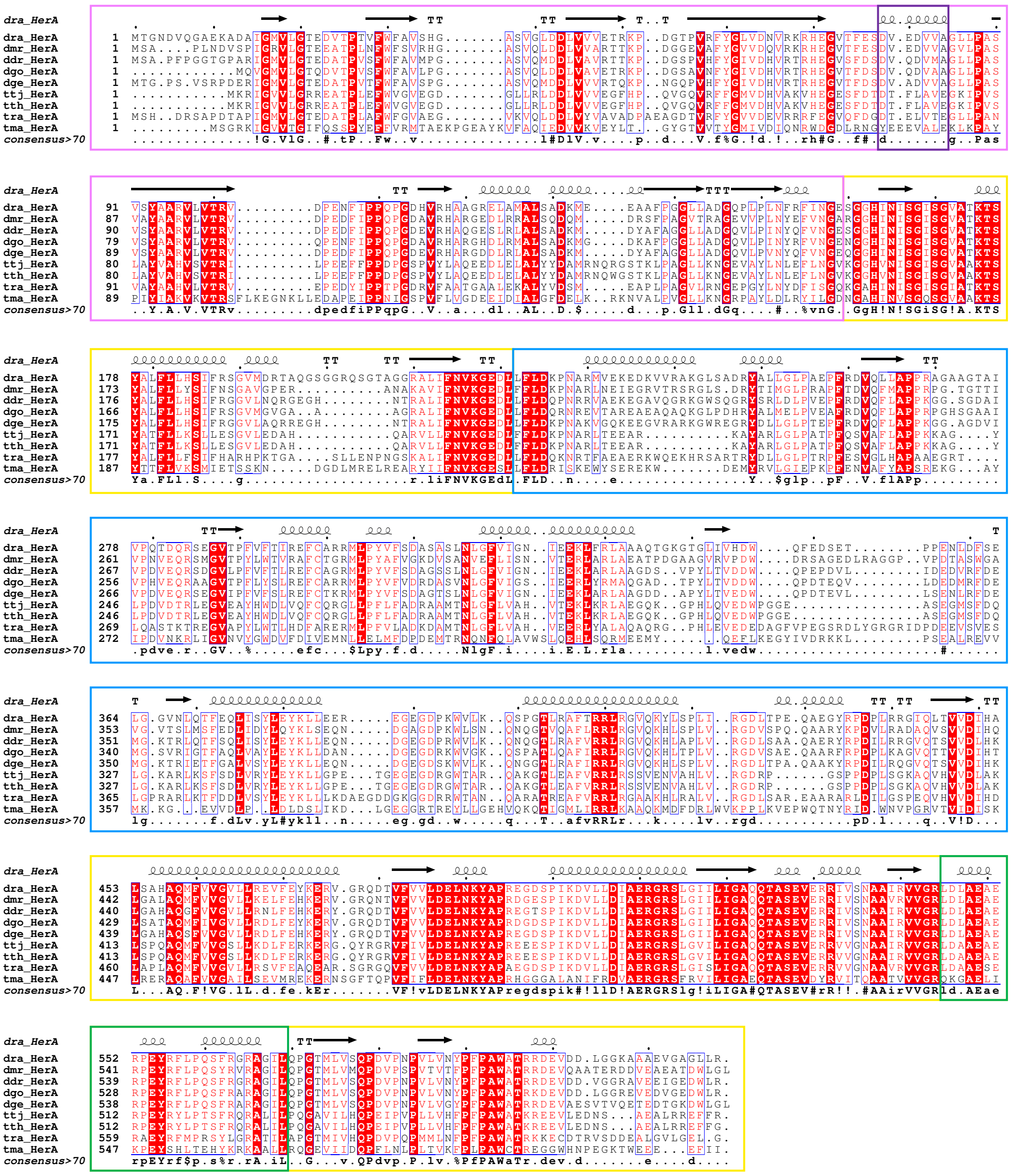

Sequence alignments of HerA proteins from different bacteria.

The HAS domain, RecA-like domain, helix-bundle domain and TR motif were framed by magenta, yellow, marine and green frames, respectively. The motif for drNurA-HerA interaction was framed with purple frame. dra, *Deinococcus radiodurans*; dmr, *Deinococcus maricopensis*; ddr, *Deinococcus deserti*; dgo, *Deinococcus gobiensis*; dge, *Deinococcus geothermalis*; ttj, *Thermus thermophilus* HB8; tth, *Thermus thermophilus* HB27; tra, *Truepera radiovictrix*; tma, *Thermotoga maritima*. Secondary structural elements are depicted according to the PDB files (dra_HerA, this study), which arrows represent β-sheet, helices represent α-helices and 'T's represent 'turn's.
