# Supplementary material for: Structural and DNA end resection study of the bacterial NurA-HerA complex
Source: BMC Biol. 2023 Feb 24;21:42. doi: 10.1186/s12915-023-01542-0 (PMC9960219; doi:10.1186/s12915-023-01542-0)
Supplement: Supplementary file 5 — Additional file 5: Figure S5. Sequence alignments of NurA proteins from different bacteria. The abbreviations of each bacterium are the same as Figure S4. The pin-like motifs were framed with cyan frame. The motifs for drNurA-HerA interaction were framed with orange frames and numbered. Secondary structural elements are depicted according to PDB files (dra_NurA, this study; tma_NurA, PDB ID=1ZUP), which arrows represent β-sheet, helices represent α-helices and ‘T’s represent ‘turn’s. [file 12915_2023_1542_MOESM5_ESM.pdf]

Additional file 5: Figure S5.

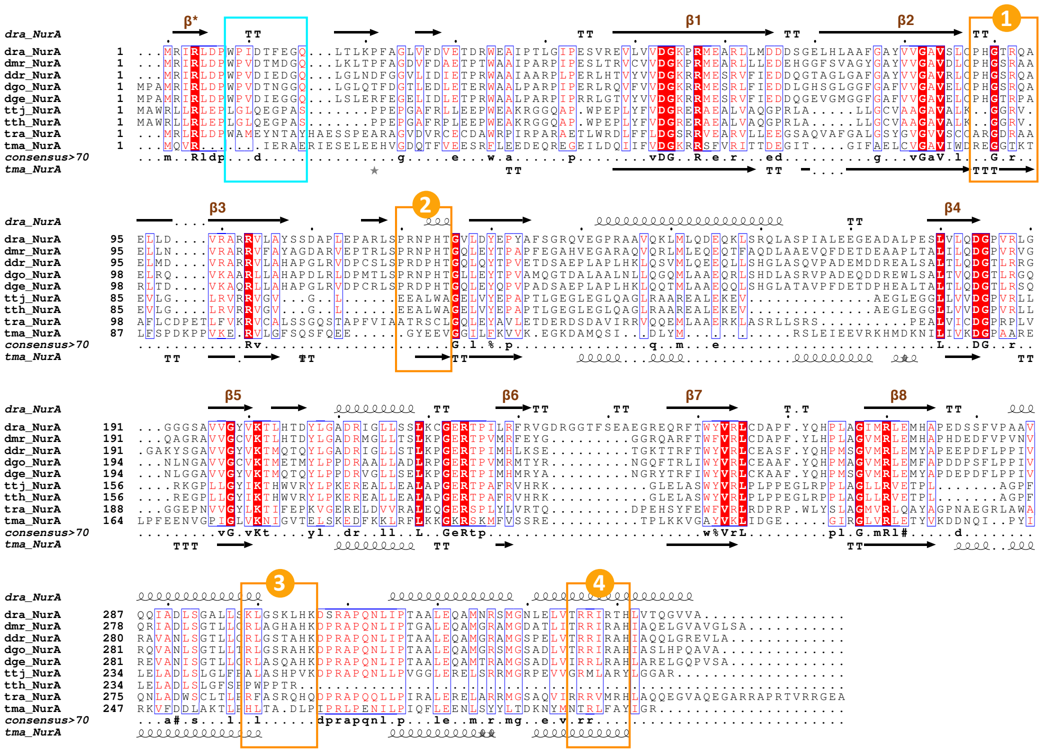

Sequence alignments of NurA proteins from different bacteria.

The abbreviations of each bacterium are the same as Supplementary Figure S4. The pin-like motifs were framed with cyan frame. The motifs for drNurA-HerA interaction were framed with orange frames and numbered. Secondary structural elements are depicted according to PDB files (dra_NurA, this study; tma_NurA, PDB ID=1ZUP), which arrows represent β-sheet, helices represent α-helices and 'T's represent 'turn's.
