# Supplementary material for: Structural and DNA end resection study of the bacterial NurA-HerA complex
Source: BMC Biol. 2023 Feb 24;21:42. doi: 10.1186/s12915-023-01542-0 (PMC9960219; doi:10.1186/s12915-023-01542-0)
Supplement: Supplementary file 6 — Additional file 6: Table S1. Oligos used in this study. [file 12915_2023_1542_MOESM6_ESM.pdf]

Additional file 6: Table S1.

Oligos used in this study.

Primers	Sequence (5′→3′)
drnurA_22FN	gaggagatatacatatgcgtattcgtctggaccc
drnurA_22RX	ggtggtggtgctcgagcgccaccaccccctgc
drherA_28FN	tacttccaaggtcatatgacggggaatgacgtgcaa
drherA_28RB	gagctcgaattcggatcctcagcgcagcagcccc
drnurA_22dN_FN	gaggagatatacatatgtggcctatcgatacttttgagg
herA_487_488A_F	ggcgtacttgttcaacgcggcgagcaccacgaacac
herA_487_488A_R	gtgttcgtggtgctcgccgcgttgaacaagtacgcc
herA_78_80_81A_F	ccgccaccacggccgccacggccgactcgaag
herA _78_80_81A_R	cttcgagtcggccgtggcggccgtggtggcgg
nurA_336_337_339A_F	cgtcaccaggtgggtggcgatggccgcggtgacgagttcgagg
nurA_336_337_339A_R	cctcgaactcgtcaccgcggccatcgccacccacctggtgacg
nurA_89_92A_F	actcggcctgcgccgtgccggcgggacacaggc
nurA_89_92A_R	gcctgtgtcccgccggcacggcgcaggccgagt
nurA_117_121_124A_F	accccggtggctgggttggctgggctgagggcggcaggttc
nurA_117_121_124A_R	gaacctgccgccctcagcccagccaacccagccaccggggt
nurA_299_303_305_306A_F	ggcgctgctaagcgcgctgggcagcgcgcttgccgcggacagccgcgc
nurA_299_303_305_306A_R	gcgcggctgtccgcggcaagcgcgctgcccagcgcgcttagcagcgcc
nurA_dePin_F	tttcagcgtcagttgaggccaggggtccag
nurA_dePin_R	ctggacccctggcctcaactgacgctgaaa
nurA_W8A_F	tcaaaagtatcgataggcgcggggtccagacgaatacg
nurA_W8A_R	cgtattcgtctggaccccgcgcctatcgatacttttga
nurA_F13A_F	gttgcccctcagcagtatcgataggccaggggtc
nurA_F13A_R	gacccctggcctatcgatactgctgaggggcaac
nurA_D53A_F	cggggtttgccggccaccaccagca
nurA_D53A_R	tgctggtggtggccggcaaaccccg
nurA_K201A_F	cggtgtgcagcgtcgcgacgtagcccacca
nurA_K201A_R	tggtgggctacgtcgcgacgctgcacaccg
nurA_R309A_F	ggttttgcggggcggcgctgtccttgtgaa
nurA_R309A_R	ttcacaaggacagcgccgccccgcaaaacc
herA_R96A_F	cgtgacgagcaccgccgcggcgtagctc
herA_R96A_R	gagctacgccgcggcggtgctcgtcacg
herA_R495A_F	gctatcaccctcggccggggcgtacttg
herA_R495A_R	caagtacgccccggccgagggtgatagc
herA_R552A_F	cggtactcgggcgcctcggcttcggc
herA_R552A_R	gccgaagccgaggcgcccgagtaccg
O1	5’Cy5_agtagaaagccaatccaccaaaaagaccctgaacgagagcctggaccccccgtccaggctctcgttcagggtctttttggtggattggctttctact_FAM-3’
O3	5’FAM_agtagaaagccaatccaccaaaaagaccctgaacgagagcctggaccccccgtccaggctctcgttcagggtctttttggtggattggctttctact
O4	5’FAM_agtagaaagccaatccaccaaaaagaccctgaacgagagcctggaccccccgtccaggctctcgttcagggtctttttggtggattggctttctact

98nt_F	ttctaagaccctgaaccactcgggaaataacaagatttcatctatgaccagtacgagcttag gttgtcctggcccgcgtgcaaaggatgacagaagca
98nt_R	tgcttctgtcatcctttgcacgcgggccaggacaacctaagctcgtactggtcatagatgaa atcttgttatttcccgagtggttcagggtcttagaa

The underlined area indicated that these bases are linked by phosphorothioate bonds, to avoid the nuclease digestion.
