# Supplementary material for: Structural and DNA end resection study of the bacterial NurA-HerA complex
Source: BMC Biol. 2023 Feb 24;21:42. doi: 10.1186/s12915-023-01542-0 (PMC9960219; doi:10.1186/s12915-023-01542-0)
Supplement: Supplementary file 7 — Additional file 7: Figure S6. The cutting pattern analysis of drNurA and drNurA-HerA of the hairpin substrate O1 in the absence of ATP. 400 nM hairpin substrate O1 (with 5′ Cy5 and 3′ FAM labeled) was incubated with 4 μM drNurA alone or drNurA-HerA complex in the presence of 2 mM MgCl2 and 8 mM MnCl2. Reactions were carried out in the absence of ATP, and incubated at 37°C for 30 min. Reactions were stopped by the stop buffer, followed by boiling at 100°C for 5 min and flash-cooling on ice for 10 min. Products were analyzed on 15% denaturing TBE-PAGE and gels were imaged at FAM fluorescent mode. Markers were created by mixing different lengths of 5′FAM-labeled DNA oligos together. [file 12915_2023_1542_MOESM7_ESM.pdf]

Additional file 7: Figure S6.

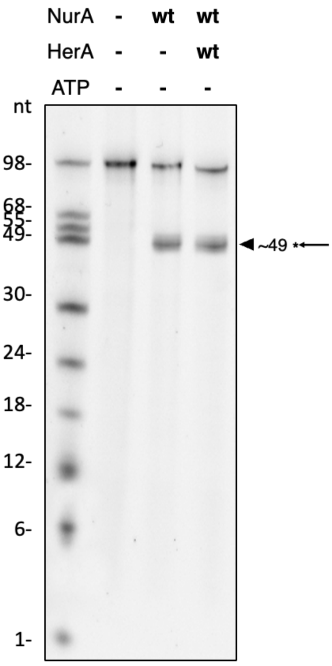

The cutting pattern analysis of drNurA and drNurA-HerA of the hairpin substrate O1 in the absence of ATP.

400 nM hairpin substrate O1 (with 5' Cy5 and 3' FAM labeled) was incubated with 4 µM drNurA alone or drNurA-HerA complex in the presence of 2 mM $MgCl_2$ and 8 mM $MnCl_2$. Reactions were carried out in the absence of ATP, and incubated at 37°C for 30 min. Reactions were stopped by the stop buffer, followed by boiling at 100°C for 5 min and flash-cooling on ice for 10 min. Products were analyzed on 15% denaturing TBE-PAGE and gels were imaged at FAM fluorescent mode. Markers were created by mixing different lengths of 5'FAM labeled DNA oligos together.
