# Supplementary material for: Structural and DNA end resection study of the bacterial NurA-HerA complex
Source: BMC Biol. 2023 Feb 24;21:42. doi: 10.1186/s12915-023-01542-0 (PMC9960219; doi:10.1186/s12915-023-01542-0)
Supplement: Supplementary file 8 — Additional file 8: Figure S7. The DNA end resection activities of His-tagged drNurA-HerA and drNurA-HerA without tag. 400 nM 5′ FAM-labeled substrate DNA (O3 or O4) was incubated with His-tagged drNurA-HerA and drNurA-HerA without tag (2 μM) in the presence of 2 mM MgCl2 and 8 mM MnCl2. Reactions were carried out in the absence or in the presence of 1 mM ATP, and incubated at 37°C for 30 min. Reactions were stopped by stop buffer, followed by boiling at 100°C for 5 min and flash-cooling on ice for 10 min. Products were analyzed on 15% denaturing TBE-PAGE and gels were imaged at FAM fluorescent mode. The types of the generated products before denaturing have been annotated at the right side of the gel to aid the interpretation of the bands on the gels. Markers were created by mixing different lengths of 5′FAM-labeled DNA oligos together. [file 12915_2023_1542_MOESM8_ESM.pdf]

Additional file 8: Figure S7.

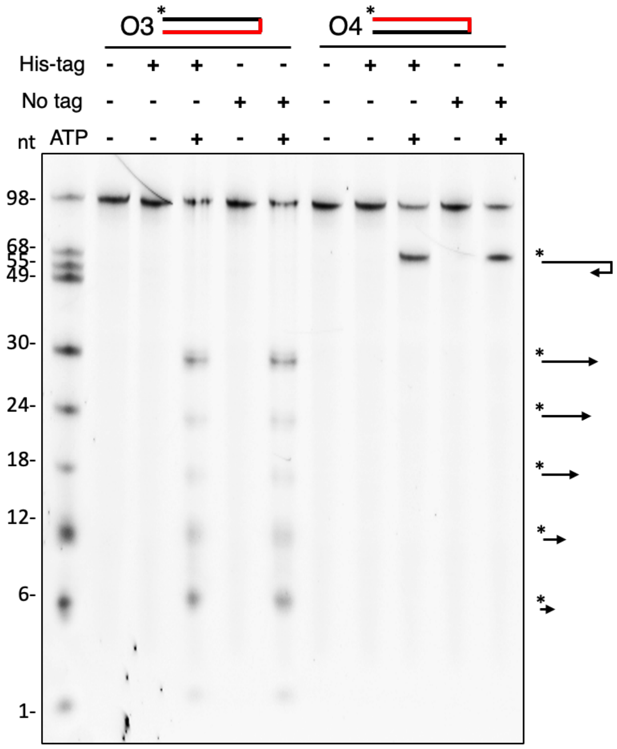

The DNA end resection activities of His-tagged drNurA-HerA and drNurA-HerA without tag. 400 nM 5' FAM labeled substrate DNA (O3 or O4) was incubated with His-tagged drNurA-HerA and drNurA-HerA without tag (2 μM) in the presence of 2 mM $MgCl_2$ and 8 mM $MnCl_2$. Reactions were carried out in the absence or in the presence of 1 mM ATP, and incubated at 37°C for 30 min. Reactions were stopped by stop buffer, followed by boiling at 100°C for 5 min and flash-cooling on ice for 10 min. Products were analyzed on 15% denaturing TBE-PAGE and gels were imaged at FAM fluorescent mode. The types of the generated products before denaturing have been annotated at the right side of the gel to aid the interpretation of the bands on the gels. Markers were created by mixing different lengths of 5'FAM labeled DNA oligos together.
