# Supplementary material for: Structural and DNA end resection study of the bacterial NurA-HerA complex
Source: BMC Biol. 2023 Feb 24;21:42. doi: 10.1186/s12915-023-01542-0 (PMC9960219; doi:10.1186/s12915-023-01542-0)
Supplement: Supplementary file 9 — Additional file 9: Figure S8. Comparisons of the ATP catalytic sites and the DNA translocation related motifs of drHerA (A), ssoHerA (B) and PaFtsK (C). Upper, zoom-in views of the ATP catalytic sites. The ligands, key residues for ATP binding and hydrolysis are shown as sticks. Middle, zoom-in views of DNA translocation related motifs. The key residues for dsDNA translocation are shown as sticks. Lower, the topology diagrams of RecA-like domain. Residues for metal binding, ATP binding, and DNA binding are shown as red, blue, and cyan dots. Insertion domains/motifs are shown as grey triangles. [file 12915_2023_1542_MOESM9_ESM.pdf]

Additional file 9: Figure S8.

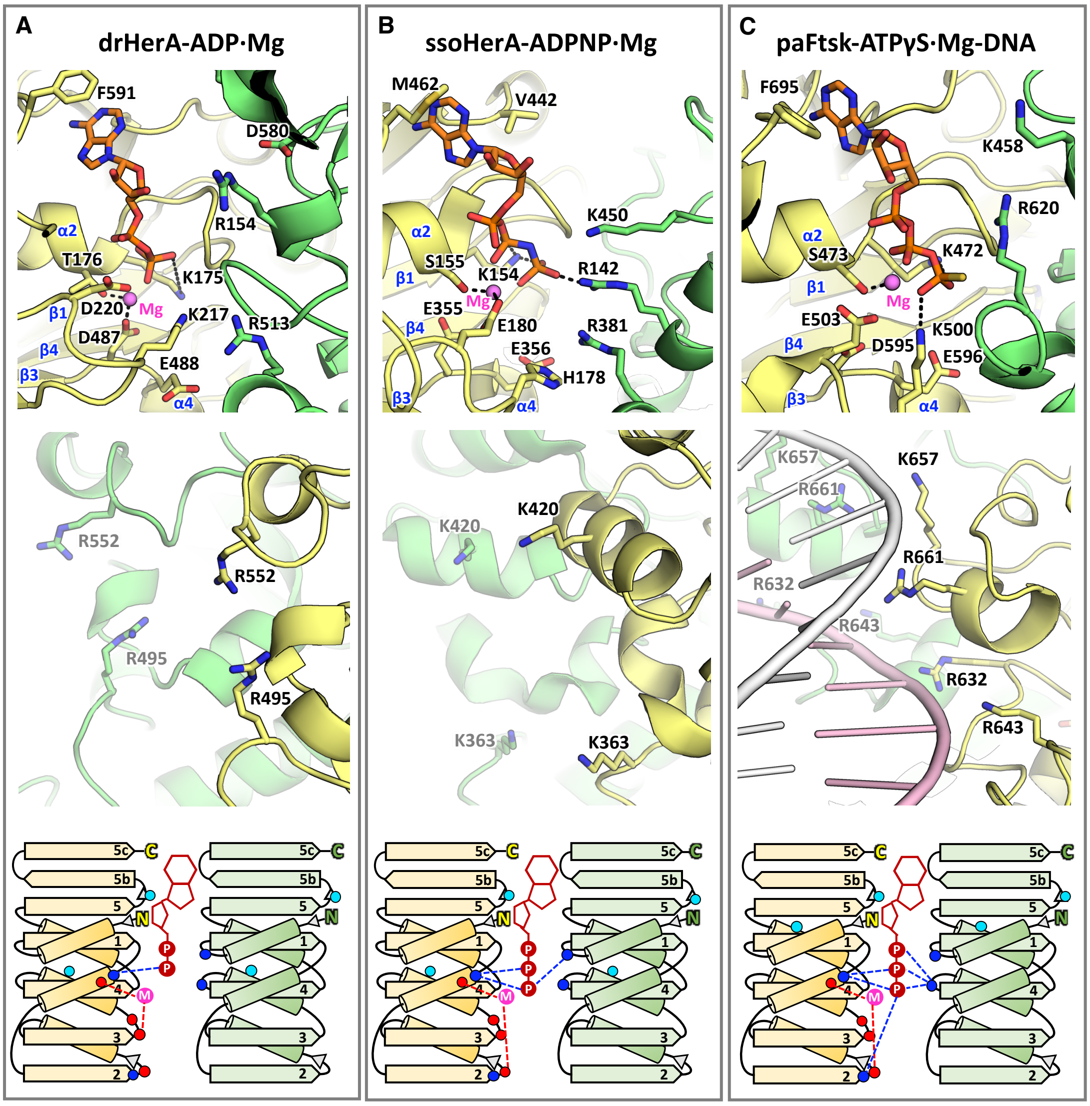

Comparisons of the ATP catalytic sites and the DNA translocation related motifs of drHerA (A), ssoHerA (B) and PaFtsK (C).

Upper, zoom in views of the ATP catalytic sites. The ligands, key residues for ATP binding and hydrolysis are shown as sticks. Middle, zoom in views of DNA translocation related motifs. The key residues for dsDNA translocation are shown as sticks. Lower, the topology diagrams of RecA-like domain. Residues for metal binding, ATP binding, and DNA binding are shown as red, blue, and cyan dots. Insertion domains/motifs are shown as grey triangles.
