# Supplementary material for: Structural and DNA end resection study of the bacterial NurA-HerA complex
Source: BMC Biol. 2023 Feb 24;21:42. doi: 10.1186/s12915-023-01542-0 (PMC9960219; doi:10.1186/s12915-023-01542-0)
Supplement: Supplementary file 10 — Additional file 10: Figure S9. The phenotypes of drNurA-HerA deletion mutant and complementation strains. Wild type strain, drNurA-HerA deletion mutant (△), drNurA-HerA complemented strain (△+nurA-herA), drNurA-HerA interaction defect mutant complemented strain (△+ nurA-herAm), the nuclease-inactive mutant complemented strain (△+nurAD53A-herA), the ATPase-inactive mutant complemented strain (△+ nurA-herAE487A/E488A), and the unwinding activity defect mutant complemented strain (△+ nurAdPin-herA) were treated with different FUV (600 J/m2) and MMC (40 μg/ml). Cells were diluted and dotted on plates. Plates were cultured for 2−3 days at 30°C. [file 12915_2023_1542_MOESM10_ESM.pdf]

Additional file 10: Figure S9.

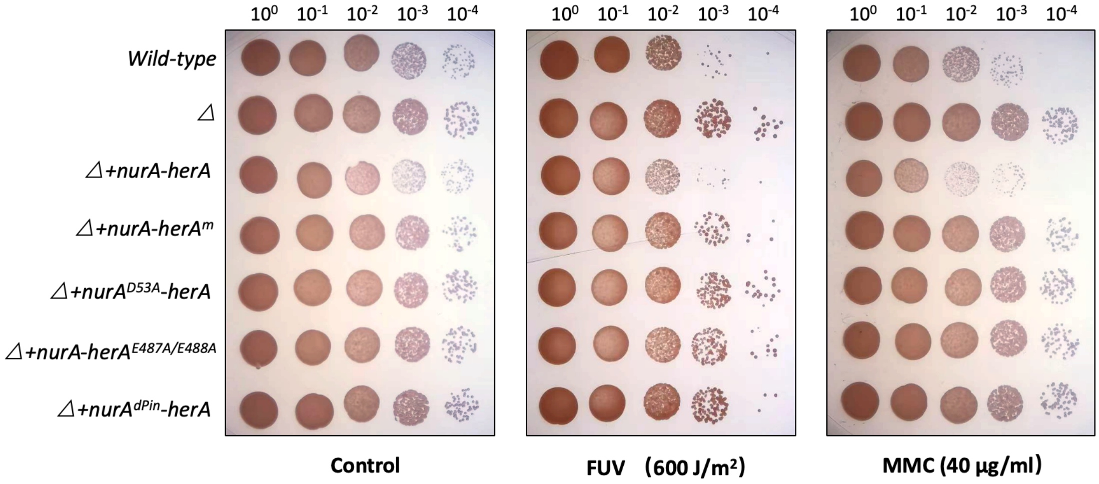

The phenotypes of drNurA-HerA deletion mutant and complementation strains.

Wild type strain, drNurA-HerA deletion mutant (△), drNurA-HerA complemented strain (△+*nurA-herA*), drNurA-HerA interaction defect mutant complemented strain (△+ *nurA-herA*^{m}), the nuclease inactive mutant complemented strain (△+*nurA*^{D53A}-*herA*), the ATPase inactive mutant complemented strain (△+ *nurA-herA*$^{E487A/E488A}$), and the unwinding activity defect mutant complemented strain (△+ *nurA*^{dPin}-*herA*) were treated with different FUV (600 J/m^2) and MMC (40 μg/ml). Cells were diluted and dotted on plates. Plates were cultured for 2−3 days at 30°C.
