# Supplementary material for: Structural and DNA end resection study of the bacterial NurA-HerA complex
Source: BMC Biol. 2023 Feb 24;21:42. doi: 10.1186/s12915-023-01542-0 (PMC9960219; doi:10.1186/s12915-023-01542-0)
Supplement: Supplementary file 11 — Additional file 11: Figure S10. The RNaseH activity analysis of drNurA and the drNurA-HerA complex. A 60 nt 5′ FAM-labeled ssRNA or 60 bp RNA/DNA duplex were incubated with 1, 2, or 4 μM drNurA dimer (in the absence or presence of 1, 2, or 4 μM drHerA hexamer), in the presence of 2 mM MgCl2 and 8 mM MnCl2 at 37°C for 30 min. Reactions were carried out using the same reaction condition as Figure 4A, in the absence or presence of 1 mM ATP. The products were resolved by 15% TBE-urea denaturing gel. [file 12915_2023_1542_MOESM11_ESM.pdf]

Additional file 11: Figure S10.

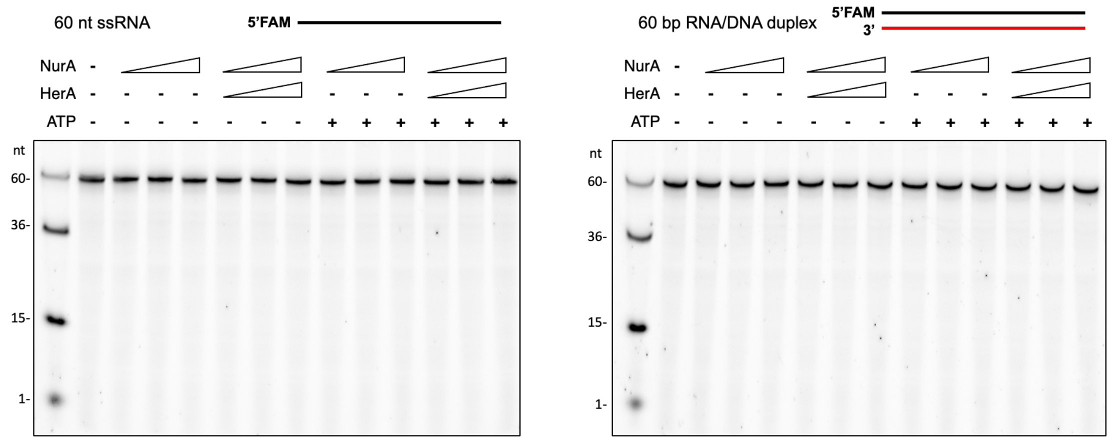

The RNaseH activity analysis of drNurA and the drNurA-HerA complex.

A 60 nt 5' FAM labeled ssRNA or 60 bp RNA/DNA duplex were incubated with 1, 2, or 4 μM drNurA dimer (in the absence or presence of 1, 2, or 4 μM drHerA hexamer), in the presence of 2 mM $MgCl_2$ and 8 mM $MnCl_2$ at 37°C for 30 min. Reactions were carried out using the same reaction condition as Figure 4A, in the absence or presence of 1 mM ATP. The products were resolved by 15% TBE-urea denaturing gel.
